# Limitations and challenges of genetic barcode quantification

**DOI:** 10.1038/srep43249

**Published:** 2017-03-03

**Authors:** Lars Thielecke, Tim Aranyossy, Andreas Dahl, Rajiv Tiwari, Ingo Roeder, Hartmut Geiger, Boris Fehse, Ingmar Glauche, Kerstin Cornils

**Affiliations:** 1 Institute for Medical Informatics and Biometry, Faculty of Medicine Carl Gustav Carus, Technische Universität Dresden, 01307 Dresden, Germany; 2 Research Dept. Cell and Gene Therapy, Department of Stem Cell Transplantation, University Medical Centre Hamburg-Eppendorf, Martinistr. 52, 20246 Hamburg, Germany; 3 Deep Sequencing Group SFB 655, Biotechnology Center, Technische Universität Dresden, 01307 Dresden, Germany; 4 Institute of Molecular Medicine and Stem Cell Aging, University of Ulm, Albert-Einstein-Allee 11c, D-89081 Ulm, Germany; 5 Division of Experimental Hematology and Cancer Biology, Cincinnati Children’s Hospital Medical Center and University of Cincinnati, Cincinnati, OH, USA

## Abstract

Genetic barcodes are increasingly used to track individual cells and to quantitatively assess their clonal contributions over time. Although barcode quantification relies entirely on counting sequencing reads, detailed studies about the method’s accuracy are still limited. We report on a systematic investigation of the relation between barcode abundance and resulting read counts after amplification and sequencing using cell-mixtures that contain barcodes with known frequencies (“miniBulks”). We evaluated the influence of protocol modifications to identify potential sources of error and elucidate possible limitations of the quantification approach. Based on these findings we designed an advanced barcode construct (BC32) to improved barcode calling and quantification, and to ensure a sensitive detection of even highly diluted barcodes. Our results emphasize the importance of using curated barcode libraries to obtain interpretable quantitative data and underline the need for rigorous analyses of any utilized barcode library in terms of reliability and reproducibility.

Reliable quantification of clonal progeny from single cells and changes in the dynamics of polyclonal patterns is critical for our understanding of tissue and cancer development, especially in stem cell driven and highly dynamic tissues like the hematopoietic system. Early efforts of cell marking via radioisotopes or dyes were successively replaced in the 1990s by the use of integrating retroviral vectors, which allowed for stable marking of cells and cell clones^1^,^2^. With the introduction of fluorescent proteins, marked cells could easily be identified and tracked^3^. However, long-term clonal-fate studies in multi-cellular tissues require a huge variety of different individual markers, which in the case of proteins need to be stably expressed^4^,^5^. To address this limitation, viral integration sites (IS) have been used as unique cell markers, relying on the semi-random nature of retroviral integration. However, IS detection and quantification using PCR-based methods strongly depend on the choice of restriction enzymes which determine actual amplicon sizes. In fact, the majority of produced DNA fragments will have various lengths which unavoidably will lead to fragment-specific amplification efficiencies and therefore to a biased quantitative information^6^,^7^. Consequently, only 60–80% of all integrations are detectable by LM- or LAM-PCR based procedures^8^,^9^,^10^.

In order to overcome those limitations, Schepers *et al*.^11^ used random nucleotide sequences of defined length within a viral carrier to mark T-cell populations. These sequences are commonly known as *cellular* or *genetic barcodes* since they equip individual cells with a unique and inheritable signature. Cellular barcodes are designed in an equally sized manner, which should allow for simultaneous amplification and an unbiased quantification of every single barcode. Although the approach by Schepers *et al*. was based on a specific microarray platform, subsequent application of a similar barcode construct to hematopoietic stem cells showed the quantitative potential of this marking strategy^12^. In particular, Gerrits *et al*. demonstrated that subcloning strategies of the barcode sequences enabled clonal analysis with unmatched sensitivity and precision. Given an appropriate sequencing depth, the barcode design should, in principle, facilitate a sufficiently high resolution for the detection of very few or even single cells within a marked cell population. As a logical next step, several other groups, including ourselves, established protocols to combine viral barcoding with next-generation sequencing (NGS)^5^,^13^,^14^,^15^,^16^,^17^ to mark and track cells not only in the field of hematopoiesis, but also in several other settings^18^,^19^,^20^. Despite their enormous potential for clonal analysis, only few reports addressed the accuracy of the quantitative results of experiments based on cellular barcodes^20^,^21^,^22^. In particular, there is still uncertainty to which extend NGS barcode read counts faithfully reproduce clonal abundances and how barcode isolation and sequencing mask a unique relationship^23^. However, the understanding of methodological constrains with the cellular barcoding technology is of utmost importance to allow for a quantitative interpretation of experimental results.

We report on a systematic investigation of barcode quantification and related limitations. To this end, we used an established barcode library^5^ and generated low-complexity cell mixtures, designated as miniBulks, consisting of four cell clones, previously barcoded and Sanger-sequenced. We analyzed discrepancies between expected and observed barcode abundances, reproducibility and corresponding variances. We evaluated the role of PCR-based barcode amplification as a source of error by sequentially reducing PCR cycle numbers and facilitating the access to the genomic template. Furthermore, we addressed the issue of barcode design by presenting an advanced barcode system with increased length. We explored how those design features influence the distinguishability and quantification of individual barcodes. Finally, a test of our advanced barcode design and the suggested protocol modifications was performed in the context of an annotated library to demonstrate the validity of the overall novel design.

## Material and Methods

### Barcode vector design and generation of vector libraries

We constructed a LeGO-G2-BC16 plasmid library, containing an eGFP as a fluorescent reporter and the GFP-BC16 as barcode sequence, according to the recently described RGB-barcode vectors^5^. For the GFP-BC32 construct, the original LeGO-vector backbone (LeGO-G2)^24^ was additionally equipped with a truncated sequence for the Illumina-Indexed-Adaptor and unique restriction enzyme recognition sites for XbaI and XhoI (LeGO-G2-BC32). The GFP-BC32 forward oligonucleotide (Poly-GFP-Barcode-Fw) consists of 32 wobble bases interspersed by fixed triplets in a defined order and of a modified version of the Illumina-Truseq Universal-Adapter-Sequence. Additionally, we included three wobble bases upstream of the barcode to prevent phasing of the Illumina system. The barcode was manufactured by TIB Molbiol. Second-strand synthesis was performed by using primer 32-BC-Poly-Rv with 500 ng of Poly-GFP-Barcode-FW. Obtained double strands were purified using Agencourt XP-beads (Beckman Coulter) and digested with XbaI and XhoI. To exclude fragmentary barcodes, a purification step via gelextraction was performed prior to ligation into the XbaI/XhoI-digested LeGO-G2-BC32 backbone. Ligation of the BC32-oligonucleotides and the vector backbone, subsequent transformation into electrocompetent cells and estimation of theoretical complexity was done as described earlier^5^.

### Preparation of miniBulks

Viral supernatant was produced from obtained plasmid libraries of the BC16 and the BC32 constructs and subsequently used for transduction of 293T cells with a low MOI to ensure one integration per cell^25^. Single GFP-expressing cell clones were sorted three days after transduction and the individual barcode sequences were amplified and sequenced via Sanger sequencing. Four different clones were chosen and equally mixed to generate so called miniBulks. We generated four biological replicates, named A1, A2, B1 and B2, to address the reproducibility of our results.

### Viral Integration Site Analysis and Digital Droplet PCR

Ligation-mediated (LM-) PCR was performed to identify the IS for each clone^26^. Additionally to the already published primers^27^, a second set of primers had to be introduced to identify the second IS in the *BC16-C* using the 5′LTR instead of the 3′LTR. IS-analysis was performed via MAVRIC^28^. We used digital droplet PCR (ddPCR, BioRad) to examine the barcode mixing ratio of the generated miniBulks. In a duplex reaction, a barcode-specific or an IS-specific fragment (using primers and FAM-labeled-probe listed in Supplementary Table S1) and a reference amplicon were amplified as described by Cornils *et al*.^5^. Vector-copy analysis by using two primer pairs, simultaneously detecting eGFP and the corresponding reference sequences was also performed as described earlier^5^.

### 1 kb fragments

1 kb fragments were generated for each barcode by PCR on genomic DNA using an IS-specific and a vector-located primer (Supplementary Table S1). Obtained fragments were gel-purified, and the concentration was determined (Qubit, Life Technologies) to calculate the amount of double-strands. 10,000 double-strands per barcode-IS-junction were then mixed to generate 1kb-fragment *miniBulks*.

### “Colored” spike-in experiments and dilution series

We produced viral supernatant from a Cer-BC32 plasmid library (analogous to the GFP-BC32), transduced HEK293T cells at low transduction rates and sorted for Cerulean-positive cells three days thereafter using a FACSAria IIIu (Becton Dickinson, Heidelberg, Germany). Sorted cells were used to construct “colored” spike-ins by mixing the four GFP-BC32 cell clones into Cerulean background. Samples containing 10%, 1%, 0.1% and 0.01% of each known clone were generated. Additionally, we spiked the same four GFP-BC32 clones into non-transduced HEK293T cells to create samples containing 10%, 1%, 0.1% and 0.01% of each known clone within a barcode-free environment.

### Barcode-amplification and next-generation sequencing

The BC16 sequences were amplified from 200 ng of genomic DNA with the following two primers, BC-PCR-FW and BC-PCR-RV_neu, using 40, 30 or 20 cycles for the first PCR. PCR products were purified using Agencourt XP-beads (Beckman Coulter) and applied to a second PCR to attach the corresponding Illumina adapters. The second PCR consisted of 25 or 10 cycles (using primers: Ill1-Tail12 and Ill2_Tail-complete). Prior to sequencing on the Illumina Hiseq2000 System, samples were indexed during 8 cycles of additional PCR with standard Illumina-primers^5^. To ensure reproducibility, we performed every PCR and the subsequent sequencing in quadruplicates.

Introduction of the Illumina-Truseq Universal-Adapter-Sequences within the BC32 vectors allowed for a single PCR step to amplify the BC32 sequences, using 200 ng of genomic DNA. In total, 40, 30 or 20 cycles of PCR were performed using the Multiplex PCR Plus Kit (Qiagen), according to manufacturer’s protocol, with 57 °C as annealing temperature, 0.2 pmol of P5-Dual-index primer/Multiplex-primer and 0.004 pmol of a bridging oligonucleotide. Every condition was executed in four replicates. PCR products were quantified using the Qubit System (Life Technologies) and mixed in equal amounts to compose the libraries, which were subsequently sequenced on the Illumina MiSeq System (single end reads of 83 bps length). MiniBulk DNA was also digested with EcoRI, a restriction enzyme that has no recognition sequence within the barcode sequences.

### Bioinformatic data extraction filter

The NGS-derived FASTQ-files were quality filtered according to the associated Phred quality score. For each sequence, an average quality score over all nucleotide positions was calculated and sequences with an average score lower than 30 were excluded. The remaining reads were scanned for the fixed triplets of the barcode, allowing for one mismatch, and then followed by the extraction of actual barcode sequences. Obtained barcodes were sorted, counted and saved in a *raw data table* with two columns: read counts and nucleotide sequence.

### Bioinformatic data visualization – Ripple Plot

The Hamming distances (HD, number of positions at which the corresponding nucleotides are different) between all detected barcodes, according to the *raw data table*, were calculated. All entries within the resulting Hamming distance matrix that were calculated to be larger than 1 were set to 0. This reduced matrix reflects only the direct neighborhood of any barcode (i.e. all barcodes with one nucleotide difference) and served as an *adjacency matrix* for a graphical representation of the relationship network (software “Gephi”, version 0.9.1). Therein, each barcode is represented as a node while the node size reflects relative reads of this particular barcode. Each edge/link indicates a one nucleotide difference between the connected barcodes/nodes^21^. The original barcodes (which were used to construct the miniBulks) were colored in red, while all remaining barcodes were color-coded according to their minimal difference to the closest of the known original barcodes. Therefore, barcodes colored in orange only differ in one nucleotide position (HD = 1), yellow barcodes differ in two positions (HD = 2) and so on.

### Error correction

Hamming-distance based error correction utilizes the *raw data table* containing read counts and nucleotide sequences. Starting with the least abundant barcode BC_small_, the Hamming distances to all other barcodes, in an increasing order of read counts, were calculated. If there was a highly similar barcode (BC_similar_, in terms of Hamming distance and according to a previously chosen threshold) the read counts of BC_small_ were added to those of BC_similar_ and the sequence of BC_small_ was dismissed. The list of barcodes was sorted for read counts and the error correction was applied from the least to the most abundant barcode. If there were more than one highly similar barcode present, the one with the smallest amount of read counts was always selected.

After a sensitivity analysis of the effect of the chosen Hamming distance threshold (Supplementary Figure S3) and given the initial Hamming distance differences of our chosen barcodes we decided to use one fourth of the total barcode length as a conservative Hamming distance limit. Therefore, the upper Hamming distance threshold for the definition of similarity was set to HD = 4 for the BC16 and HD = 8 for BC32. The definition of such a threshold accounts for a successively increasing amount of single nucleotide mutations due to several applied PCR cycles, a potential loss of sequences due to the fact that only a part of the PCR material will finally be sequenced and errors finally introduced by NGS. The exact value of this threshold should be adapted according to the used barcode construct, the number of initially marked cells and the particular library complexity.

### Generation of annotated barcode library (ABC-library)

After the substitution of the SFFV promotor by the EFS promoter, we generated the EFS-GFP-BC32 plasmid library as described for the GFP-BC32 construct. In order to establish a library with annotated barcodes that exhibit large differences in terms of their Hamming distances, we initially picked 672 single bacterial clones after transformation and analyzed them individually using Sanger sequencing. Only barcodes with correct backbones and error-free index adapter sequences were chosen to be included into the curated library. We selected a set of 343 barcodes with a minimal Hamming distance of 12 (HD > = 12) to any other selected barcode. Corresponding plasmids were combined into five stocks, containing 68 or 69 different barcodes. After transformation and plasmid preparation, we combined the five maxi-preparations to generate the final annotated barcode library. As a quality control of the different steps, we performed Illumina-Sequencing of 10^10^ plasmids of the five stocks, the subsequently generated maxi preparations and the entire ABC-library (Supplementary Figure S10).

## Results

### Barcodes with 16 variable bases (BC16)

**Figure 1:**
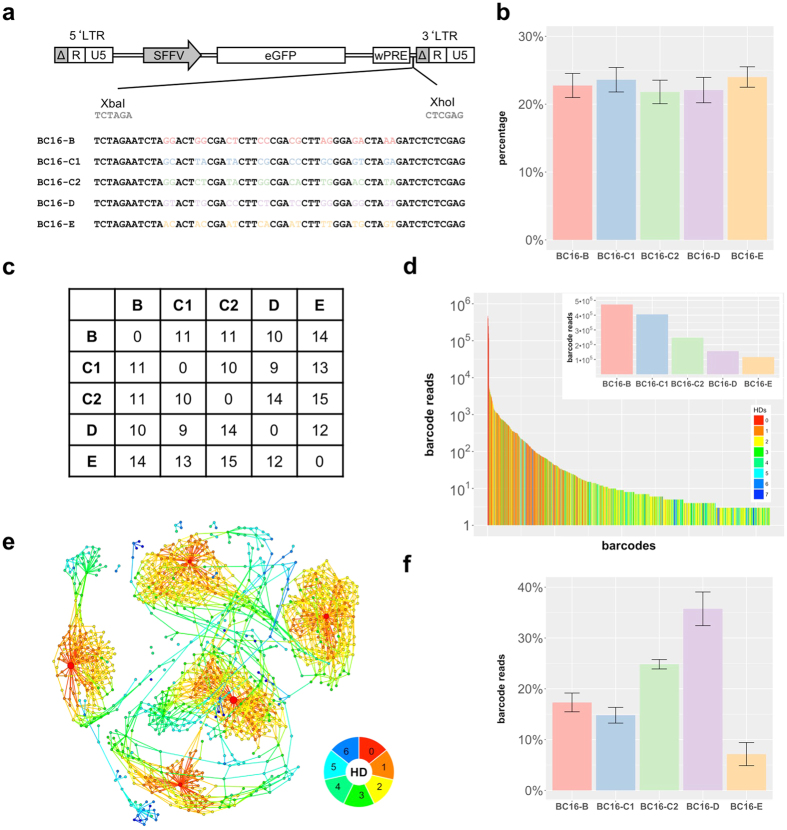
BC16 construct. (**a**) BC16 barcodes were introduced in front of the 3′ LTR of the LeGO vector. They consist of 16 random nucleotides separated by fixed triplets. (**b**) Results of the quantitative digital droplet PCR (ddPCR) which was performed directly after generating the miniBulks. (**c**) Overview of the minimal HD of the picked barcodes. (**d**) The frequency plot of one particular miniBulk depicts all identified barcodes (on a log scale) with more than two reads in a descending order. Every barcode is coloured according to its minimal HD to one of the original barcodes. The frequencies of the original five barcodes are depicted in the inset. (**e**) Sequence similarities visualized with a network based graph (ripple plot). Barcodes are represented as nodes, sequence similarities of HD = 1 between two barcodes are visualized as links and node sizes reflect read counts. The minimal HD of each barcode to one of the original barcodes is again color-coded according to the depicted legend. (**f**) The average abundance of all original barcodes over all replicates after 65 cycles of PCR.

We generated low complexity cell mixtures containing four cell clones with distinct barcodes, referred to as miniBulks. Barcode identity was established via Sanger sequencing (Fig. 1a). Due to a double integration within one clone, verified by copy number analyses, the generated miniBulks consist of four cell clones, but five different barcode sequences. Equal frequencies of all four mixed clones were verified by ddPCR directly after miniBulk generation (Fig. 1b). The identified barcodes possess a minimal Hamming distance (HD, the number of nucleotide differences between any two barcodes) of at least nine nucleotides (Fig. 1c).

To address the question about reliable and reproducible quantification, our miniBulks underwent repeated analyses (i.e. biological and technical replicates). On average, 5 × 10^5^ reads per sample were analyzed, and >72% of the raw data reads passed the filtering step, for which we verified the overall consistency of the fixed triplet positions (barcode backbone) allowing for one mismatch. Figure 1d shows the frequencies of identified barcodes (on a log scale) in a descending order for one particular miniBulk. As expected, the five original barcodes are most abundant and were detected with at least 100,000 reads each (total 91% of all reads). However, besides the five expected barcodes we also observed a large number of false-positive barcodes. Many of these had sequences closely similar to those of the original barcodes, indicating that these false-positive ones are deviating from the original sequences. This deviation might be a consequence of erroneous PCR amplification or errors during NGS analysis. We refer to these deviating barcode sequences as *descendent barcodes*.

Frequency distribution in Fig. 1d shows that descendent barcodes with a lower Hamming distance to the original sequences are more frequent than descendent barcodes with greater Hamming distances. This suggests that descending barcodes might also result from secondary or tertiary accumulation of PCR and/or sequencing errors. A convenient method to depict this relationship is the use of network graphs, in which barcodes are represented as nodes and sequence similarities as links. Figure 1e shows a representation in which links represent only sequence differences of one nucleotide (HD = 1) between the two connected barcodes. The typical concentric circle-like structures (*ripples*) illustrate the notion of original and descendent barcodes. Every original barcode (red nodes in the center) is accompanied by at least one layer of descendent barcodes arranged like water ripples caused by one single event. Each ripple can lead to another ripple by subsequently introduced sequence errors. Hamming distances to one of the original barcodes are again color-coded.

Interestingly, the initially even distribution of the five original barcodes was not retained during the analysis (Fig. 1d). Relative abundances of the five original barcodes for independent sequencing runs of the four biological replicates are shown in Fig. 1f. Although we obtained reproducible patterns, barcodes were not equally represented in the analysis. This was in obvious contrast to the ddPCR data indicating an even distribution of the four clones. In fact, we observed that read counts for barcode *BC16-E* always contributed less than the expected 20% and *BC16-D* was predominantly overrepresented. Surprisingly, *BC16-C1* and *BC16-C2*, both derived from the same clone, were also reproducibly measured with unequal read counts, strongly suggesting a quantification bias within the applied methods.

Given the close similarity in the overall barcode sequences with identical backbone structures and flanking regions, we could not initially rule out that unspecific hybridization of unfinished PCR products biases our results. However, we explicitly checked for such hybrid sequences by scanning the obtained sequence reads for “chimeric” barcodes, but detected them only in spurious fractions. We conclude that unspecific hybridization has only a minor effect on the quantitative bias.

#### Influence of PCR polymerase and cycle numbers

We next investigated the influence of the applied PCR protocol on the quantitative bias and the frequency of descent barcodes. Initially, the established standard PCR protocol (65 cycles of PCR in combination with a standard Taq polymerase, see Material and Methods) resulted not only in a skewed frequency distribution of the original barcodes but also in a substantial amount of highly similar descendent barcodes (low Hamming distances). Figure 1e illustrates this effect by the frequency of connections between the distinct (original) barcodes and their descendants. Consequently, we investigated the extent to which a change of polymerase and the total number of PCR cycles influences both the quantitative bias and the frequency of descent barcodes.

First, we asked whether the use of a proofreading polymerase for the PCR amplification would reduce the overall error rate and lead to a decrease in the number of descendent barcodes. To do so, we replaced the Taq polymerase from the Multiplex Plus PCR Kit (Qiagen) by the Q5 High-Fidelity DNA Polymerase (New England Biolabs) with a supposedly over 100 times higher fidelity^29^. Unexpectedly, the number of descendent barcodes did not decrease, and also the proportions of descendent barcodes did not change considerably between the two experimental setups.

**Figure 2:**
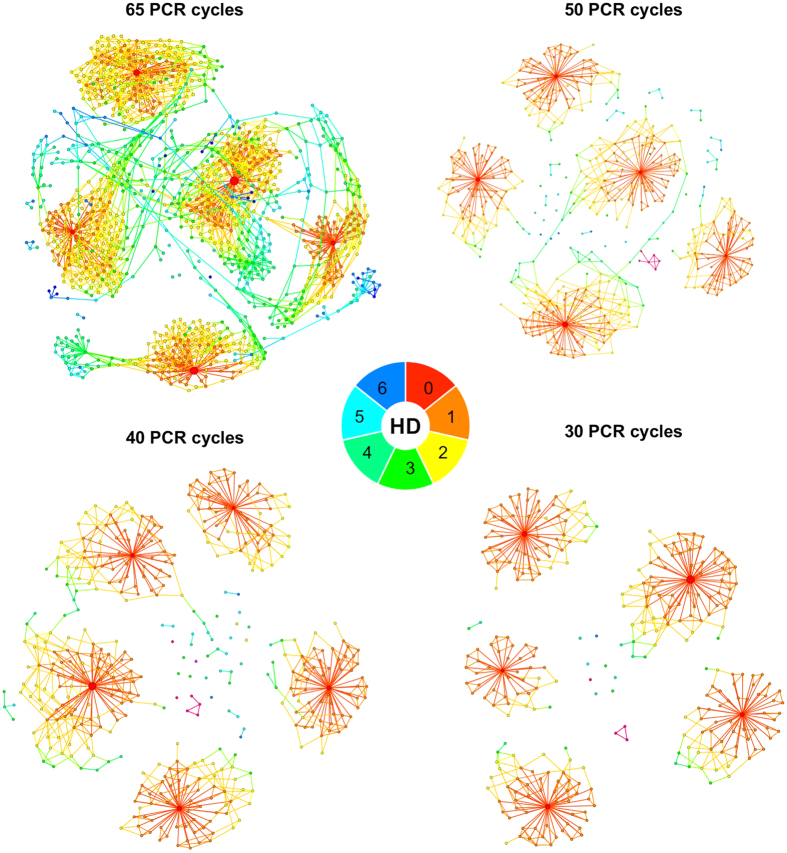
Influence of different PCR-cycle quantities – BC16. Influence of a decreasing number of PCR cycles on the generation of descendent barcodes visualized as a network based graph (ripple plot). Barcodes are represented as nodes, sequence similarities with a HD = 1 are visualized as links and node sizes reflect read counts. The minimal nucleotide differences between each barcode and one of the original barcodes is color-coded as shown in the legend. Original barcodes are “red” (HD = 0), descended barcodes with a HD = 1 are “orange” and so on as depicted in the color scale. Shown is a PCR cycle reduction without restriction enzyme treatment.

Next, we focused on the total number of PCR cycles and their influence on not only the number of descendent barcodes, but also on the distribution of read counts of all five original barcodes. We addressed this effect by gradually decreasing the PCR cycle numbers from 65 to 30 cycles in our four replicates. In particular, we reduced cycle numbers within the first PCR from 40 to 20 and within the second from 25 to 10 cycles. Graphic analyses of the obtained BC16 data via ripple plots visualizes the benefit of PCR cycle reduction. We clearly observed a pronounced reduction of descended barcodes and also a decreasing number of connections between the five barcode clusters, which eventually even disappeared, exemplarily shown as ripple plots of BC16 data in Fig. 2. The reduced ambiguity with respect to the origin of the descendent barcodes substantially improves the applied bioinformatical error-correcting methods.

**Figure 3:**
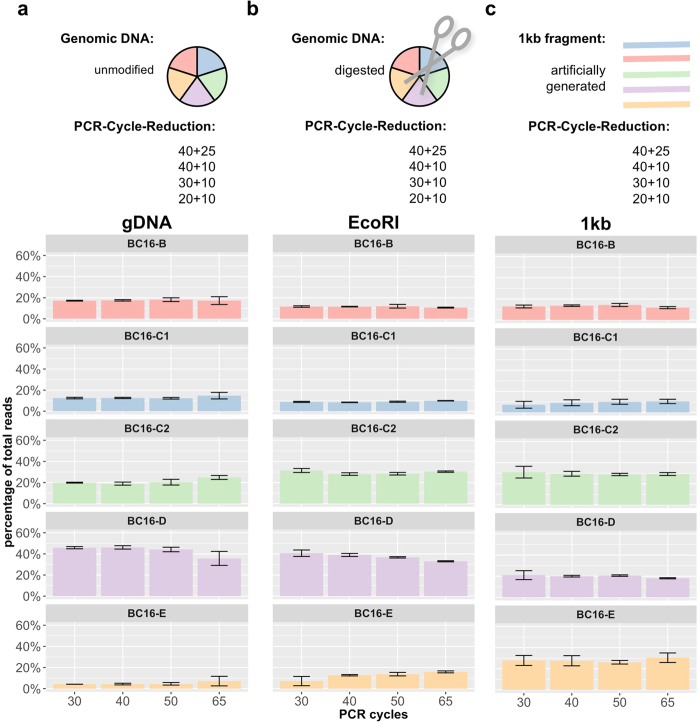
PCR protocol modifications – BC16. Every barcode mixture was analysed using a successive reduction of PCR cycle numbers from 65 down to 30 PCR cycles in combination with different sample preparation strategies, namely the (**a**) standard protocol (gDNA), (**b**) an improved protocol with an additional restriction digest (EcoRI) and (**c**) a totally artificial protocol consisting of previously build and afterwards mixed 1 kb long DNA fragments (1 kb). The relative average barcode abundances of all miniBulk replicates are shown as barplots.

Surprisingly, while the frequency of the descendent barcodes could be reduced by decreasing PCR cycle numbers, the overall bias in the relative frequency of the original barcodes was not altered. Barplots of four independent biological replicates, shown in Fig. 3a, reveal the unbalanced but at the same time reproducibly biased distribution of barcode frequencies. Conservation of the order of barcode abundances (highest: *BC16-D*, lowest: *BC16-E*) support the existence of an underlying systematic bias.

#### Accessibility of barcode sequences

The limited influence of the PCR protocol changes left us with the question whether the accessibility of the barcode sequence within its chromosomal surrounding might be a determining factor of the quantification bias. As identified via LM-PCR (Supplementary Figure S4), each of the five proviruses that marked the four cell clones integrated into different chromosomes. In order to improve primer access to the barcode sequences, we digested the genomic DNA with the restriction enzyme EcoRI prior to the first PCR amplification step. This particular enzyme was chosen to prevent cutting within the barcode sequence. Digested DNA was used as template for amplification, again with a gradually decreasing number of PCR cycles in quadruplicates, as we did for the undigested miniBulks (Fig. 3b). Although the procedure resulted in higher read counts for *BC16-C2* and *BC16-E* in comparison to the non-digested samples, a change in the overall pattern could not be observed.

To further increase the access to the integrated barcode sequences, we generated fragments of 1 kb size from the provirus-genome junction that included the barcode sequences (Supplementary Figure S4). For each fragment, 10,000 molecules were mixed together and taken as templates for amplification. As outlined in Fig. 3c, all experiments were again performed with PCR cycle reduction and the results of four independent experiments are reported. Although this kind of template should be the most accessible and it also led to higher read counts of *BC16-E*, the overall bias of the barcode abundances remained.

### Barcodes with 32 variable bases

#### Improving the BC structure

For a barcode construct with 16 wobble bases, there are 4^16^ possible nucleotide combinations leading to 4.3 × 10^9^ theoretically possible barcodes. However, picking 10,000 barcodes randomly from a BC16 library, the average Hamming distance of the randomly selected barcodes is HD = 4.7 (given uniformly distributed nucleotides). As demonstrated for the BC16 construct, close sequence similarities and regular occurrence of a substantial amount of descendent barcodes will unavoidably limit the identifiability of the original barcode sequences. To overcome this limitation, we developed a new version of our barcode construct, for which we doubled the number of wobble bases from 16 to 32. Simulation studies showed that for barcode designs of such length the average Hamming distance of 10,000 randomly picked barcodes will be increased to HD = 13.8 (Supplementary Figure S1a).

**Figure 4:**
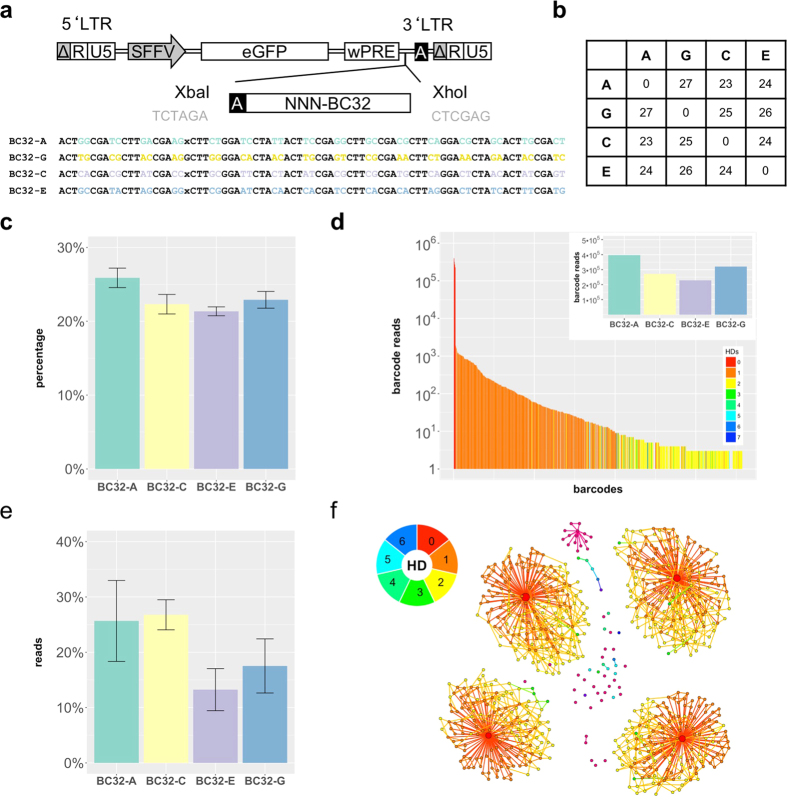
BC32 construct. (**a**) The LeGO-vectors were additionally equipped with the second Illumina adaptor and dedicated restriction enzyme recognition sites. The barcodes consist of 32 random nucleotides separated by fixed triplets. (**b**) Overview of the minimal HD of the picked barcodes. (**c**) Quantitative ddPCR based on integration sites was performed directly after generating the miniBulks. (**d**) The frequency plot of one particular miniBulk depicts all identified barcodes with more than two reads (log scale). Every barcode is colored according to its minimal HD to one of the five original barcodes. The original four barcodes are depicted in the inset. (**e**) The average abundance of all four original barcodes over all miniBulk replicates after 40 cycles of PCR (standard protocol, gDNA). (**f**) Sequence similarities are visualized with a network based graph (ripple plot). Barcodes are represented as nodes, sequence similarities of a HD = 1 between two barcodes are visualized as links and node sizes reflect read counts. The minimal HD of each barcode to one of the original barcodes is color-coded according to the depicted legend.

We also designed a new plasmid backbone that already contains a truncated version of the index-adaptor sequence within the viral backbone. The adaptor sequence was introduced in direct conjunction with the barcode construct (Fig. 4a). Hence, the amplification of the genomic DNA, as well as the introduction of Illumina-indices for multiplexing can now be performed in one single PCR step (Supplementary Figure S1b) which significantly decreases the required number of PCR cycles necessary to obtain a sequencing-ready product, which further reduce the number of derivatives.

To evaluate our new BC32 construct, we generated artificial low-complexity bulks by selecting four barcoded cell clones with a minimal Hamming distance of 23 (Fig. 4b) and by combining them in equal amounts to obtain miniBulks, as described for the BC16 construct. Verification of equal mixture ratios was again performed via ddPCR (Fig. 4c). We analyzed the generated miniBulks (four biological replicates) in accordance to the BC16 miniBulks. The results revealed that the obtained BC32 read counts were still slightly biased but less, compared to the BC16 barcodes. Frequencies and amounts of the acquired mutations in descendent barcodes were also clearly reduced (Fig. 4d). *BC32-A* and *BC32-C* showed an abundance of around 25% as expected. *BC32-G*, depending on the protocol, was either over- or underrepresented and *BC32-E* was constantly underrepresented (Fig. 4e). Apart from the quantification, the newly designed BC32 construct has a strong advantage concerning descent barcode cluster morphology and distribution. In fact, due to the increased Hamming distances between the selected barcodes, the clusters of descendent barcodes did no longer overlap (Fig. 4f) which leads to an increase in the ratio of read counts obtained from the original barcodes versus their related descendent barcodes (Supplementary Figure S8). Those two features are of outmost importance for barcode calling especially in situations, in which the original barcodes are not known and need to be identified based on overrepresented read counts and unambiguously associated descendent barcodes^30^.

#### PCR cycle reduction and access to genomic integration site

**Figure 5:**
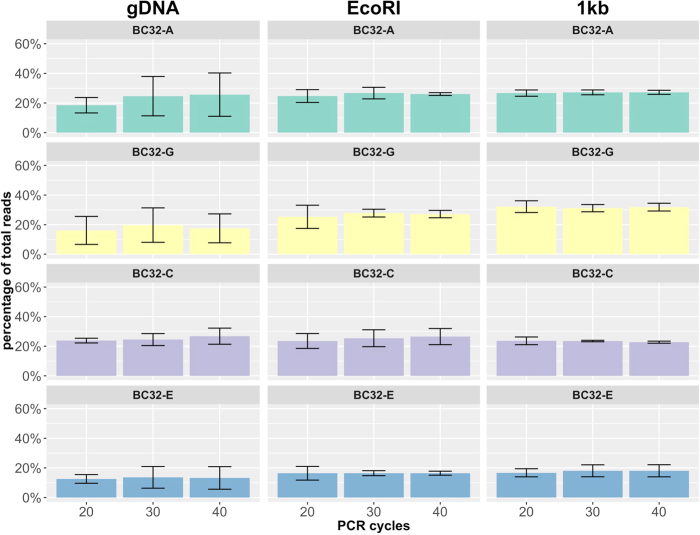
PCR protocol modifications – BC32. Every barcode mixture was analysed using a successive reduction of PCR cycle numbers from 40 down to 20 PCR cycles in combination with different sample preparation strategies, namely the standard protocol (gDNA), an improved protocol with an additional restriction digest (EcoRI) and a totally artificial protocol consisting of previously build and afterwards mixed 1 kb long DNA fragments (1 kb). Depicted are the average barcode abundances including their standard deviation of all miniBulk replicates.

As for the BC16 construct, we performed PCR-cycle reduction experiments for the BC32 construct and applied 40, 30 and 20 cycles with four biological replicates each (Fig. 5). We also tested if the unbalanced read counts were caused by the different integration sites of the proviruses within the four miniBulk clones. In order to estimate the influence of the integration site in terms of accessibility of the PCR-target sequence, we again digested the genomic DNA with EcoRI prior to the PCR procedure and, we generated 1 kb fragments as described for the BC16 construct (Fig. 5). We also performed four technical replicates of each condition to ensure reproducibility. Independent of the parameters chosen, all four barcodes still showed an unchanged abundance.

#### Spike-In experiments and dilution series of barcoded clones

Clearly, an oligoclonal situation with a tissue comprised of four to five marked cell clones is relatively unlikely and comparatively easy to analyze. In contrast, many experimental settings aim to detect and quantify clones that contribute only to minor frequencies at a given time point. To address this problem, we used the four BC32 cell clones and mixed them in a dilution series into (i) non-transduced and (ii) barcode-transduced (Cer-BC32) HEK293T cells. We obtained mixtures containing 10%, 1%, 0.1% and 0.01% of each clone.

**Figure 6:**
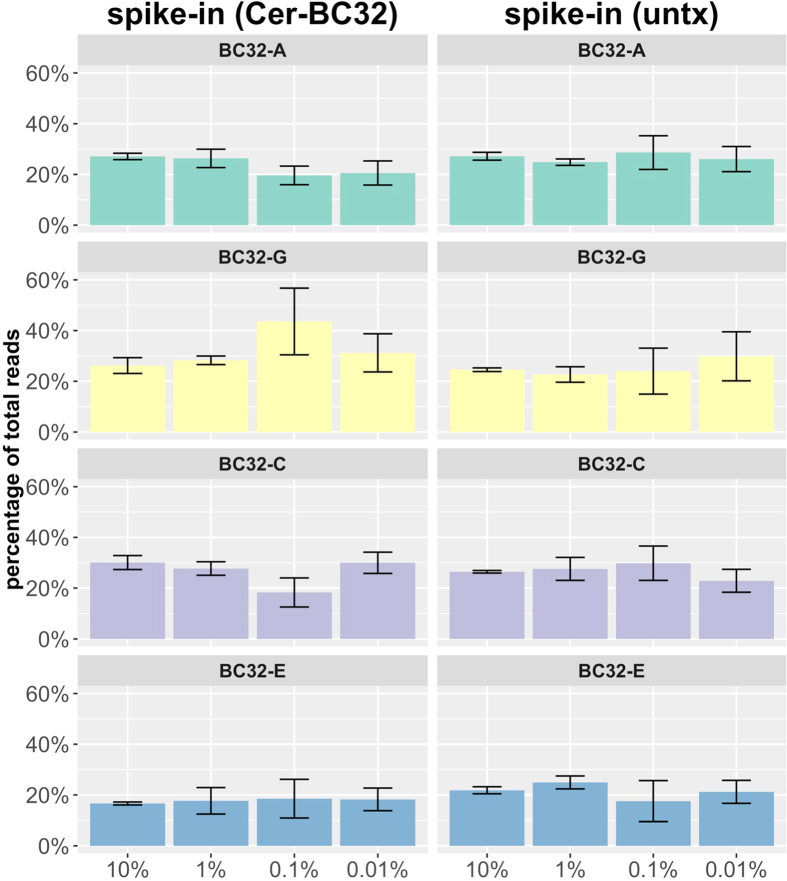
Spike-in experiments – BC32. In a dilution series, the four BC32 cell clones are mixed into non-transduced (untx) and also barcode-transduced HEK293T cells (Cer-BC32) to obtain mixtures containing 10%, 1%, 0.1% and 0.01% of each clone. The average barcode abundances in the replicates are visualized as barplots including their standard deviation. Only the relative read counts of the four original barcodes are analysed.

We processed four replicates of each mixture sample with an EcoRI-digestion and 30 cycles of PCR prior to NGS. Although, the contribution of the single clones is quite low in the 0.01% sample (Fig. 6), we obtained a reasonable number of read counts for analysis after filtering. By comparing these results to the miniBulks, we did not observe any significant differences in the read counts of the original barcodes. All four barcodes showed an almost equal contribution, even when amplified from only a small amount of marked cells within both the non-transduced and transduced setting. Taking into account that an aliquot of approx. 30,000 cells (200ng genomic DNA) was used for the analyses, we could clearly and reproducibly detect barcodes that were present in only three cells of this sample.

### ABC-Library (BC32)

**Figure 7:**
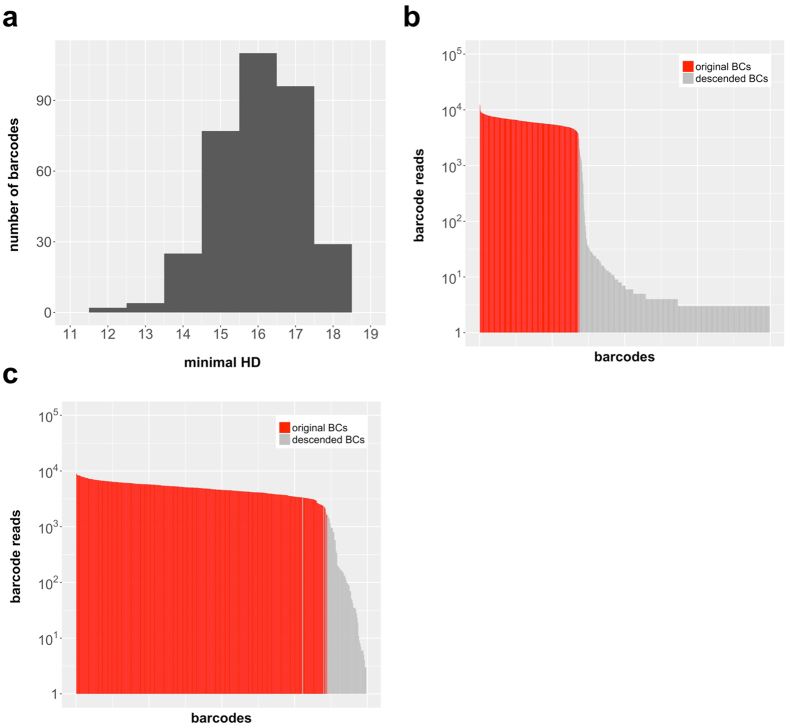
ABC-library. **(a)** Minimal HD distribution of the chosen barcodes of the annotated barcode library (ABC library). (**b**) Sequenced plasmid library shows balanced barcode frequencies and 341 of 343 barcodes in total. **(c)** Sequencing results of a barcode transduced test batch of 3T3 cells also show balanced barcode frequencies and again 341 of 343 barcodes in total.

Identifiability and quantification of individual barcodes (and their descendants) depends strongly on their mutual dissimilarity. To this end, we designed a curated BC32 library in which constituting barcodes were individually chosen based on their Hamming distance to optimize the overall dissimilarity. Technically, we expanded 672 single plasmids in seven 96 well plates of which 666 wells could by sequenced and analyzed. To ensure optimal conditions, we excluded plasmids with erroneous backbones, imperfect index adapter sequences and not entirely correct sequenced barcode sequences. Moreover, barcode plasmids selected were required to differ in at least 12 positions from any other selected barcode plasmid (Fig. 7a). Due to the selection process based on clonal pre-sequencing, the curated library (Supplementary Table S2) should fulfill all the above listed rigorously tested quality standards. By applying our developed error-correction model to the obtained NGS results of our library we achieved a sensitivity of 1.0 and a specificity of 0.96.

Validation experiments for our ABC-library, utilizing *in vitro* labeled 3T3 cells, demonstrated balanced barcode frequencies and revealed that 341 of the 343 barcodes were recovered after transduction and cell culture (Fig. 7b,c). Such an in-detail annotated and quality selected barcode library, although of moderate size, will be sufficiently complex for a large variety of labeling and clonal tracking experiments.

## Discussion

Genetic barcodes were primarily developed as a quantitative tracking method for the analyses of clonal developments, especially in the hematopoietic system. However, detailed analysis about the reliability and reproducibility of clone size quantification based on sequencing reads are still limited. Furthermore, the vast variety of published barcode constructs and their corresponding proprietary analysis protocols do not allow for a direct comparison, and a gold standard for the analysis of barcode data was never established. Even the few published validation experiments followed no standardized methods let alone are based on the use of the same type of biological material. Whereas some labs focussed entirely on the analysis of barcode equipped plasmids^20^,^23^, or a quantification of barcoded plasmids spiked into genomic DNA^31^, others used unequal mixtures entirely composed of cell clones with known barcodes^20^,^32^ or spiked-in known barcoded clones into genomic material^18^. None of these approaches can be considered as incorrect or carelessly performed but a concise standard method is still missing.

We established defined mixtures of barcodes, so called miniBulks, to systematically analyse the accuracy and sensitivity of our barcode detection protocols. Our reproducible results successfully showed that protocol changes and optimized barcode design strongly influence the quality of the readout, and furthermore the interpretation of the obtained results. Our findings also indicate that faithful reproduction of initial barcode abundances solely by counting sequencing reads obtained from NGS is still a challenging endeavor that is limited by systematic biases.

Surprisingly, neither technical variations (e.g., reduction of PCR-cycle numbers, usage of a proof-reading polymerase, prior restriction of genomic DNA using an endonuclease), nor the application of an error-correction model could sufficiently reveal or erase the discrepancies within the barcode abundances for our BC16 construct. In fact, the contributions of the five BC16 barcodes in our experiments appear systematically and reproducibly biased (Figs 1, 2, 3). However, analyzing read-count ratios of the double integrated and therefore inherently coupled *BC16-C1* and *BC16-C2* did not confirm the impression of a systematic bias, as none of the two barcodes is consistently detected with higher abundance (Supplementary Figure S6).

In order to address the question whether a different barcode design is more appropriate to overcome such limitations, we developed a new BC32 design with increased length, different barcode backbone and already integrated Illumina-Adaptor sequences (Fig. 4a). The new design resulted in a reduction of the systematic bias compared to the BC16 construct, although we still observed reproducible shifts in the barcode abundances that could not be entirely resolved by protocol modifications.

Speculating about possible reasons for the moderate but seemingly systematic bias, two obvious sources should be considered: PCR amplification of the barcodes from genomic DNA and NGS.

Regarding the PCR, it is well known that the nucleotide structure, most prominently the GC-content, influences the uniform amplification. Although we identified minor differences in the GC-content of the barcodes in question (Supplementary Figure S9), we did not observe a correlation between barcode abundance and GC-content. These findings are also consistent with previously published results of Deakin *et al*.^23^.

We also analyzed how the number of amplification steps influences the abundances of descendent barcodes. Supplementary Figure S7 depicts the relation between read counts of the original barcodes and the summed up read counts of all their respective descendent barcodes for all miniBulk experiments. The observable almost linear relationship between read counts of the original barcodes and the sum of the read counts of their corresponding descendent barcodes suggests an increasing generation of descendent barcodes along with an increasing number of PCR cycles. Consequently, it is recommendable to avoid unnecessary PCR cycles to minimize the generation of descendent barcodes, especially if there is only limited or even no prior knowledge of the deployed barcodes. Analysis of the miniBulks after different dilution steps indicates that also after a significantly reduced number of PCR cycles the achieved sensitivity is still sufficient. In fact, we could reliably and reproducibly detect barcode sequences even at a very low level of 0.01% within the samples.

Furthermore, a detailed inspection of the BC16 mutation patterns on the level of single-nucleotide substitutions revealed an additional systematic bias. By analyzing only the most similar descendent barcodes (with a HD = 1 and HD = 2 to the original barcodes) we observed a clear preference for certain nucleotide substitutions, such as A − > G (Supplementary Figure S5). These exchanges between purine or pyrimidine bases are called *transitions* and most likely result from substitutions during replication processes using a standard Taq polymerase^33^. Consequently, we also analyzed the BC16 design utilizing a Q5 High-Fidelity DNA Polymerase. Although, the frequency of transition events was reduced, the results did not notably change (Supplementary Figure S5). We also observed similar patterns for the BC32 construct although the transitions were more homogenous. Surprisingly, read counts associated to those “highly similar descendent barcodes” ranged from single reads to more than 10^5^ reads, indicating that at least some of them must have been generated very early during PCR amplification (Supplementary Figure S6). Therefore, such descendent barcodes are almost impossible to exclude only by applying a read-count threshold. Prior knowledge about the initial barcodes is one of the most promising strategies to circumvent this problem.

In conjunction with bioinformatical approaches to deal with generated PCR artifacts, a promising alternative to a curated library with known barcodes has to be considered. Unique molecular identifiers (UMIs) present a comparatively new approach to cope with the problem of a quantitative amplification biases^34^,^35^,^36^,^37^. By individually labeling all PCR templates prior to the actual amplification process, template specific amplification probabilities should, in theory, be resolvable by counting unique UMIs in the end. Therefore, UMIs represent a promising alternative to curated barcode libraries especially in the context of *in vivo* generated barcode constructs^30^,^35^.

Although we primarily focused on errors during PCR amplification, NGS represents the second possible source of bias. This appears even more likely for “low-diversity sequencing” as it is the case for the presented barcode constructs with the fixed backbone structure. Nucleotide diversity is essential for an effective template generation and subsequent reliable sequencing on several sequencing platforms including Illumina. We aimed to circumvent those problems by (i) spiking a high diversity library (PhiX) into the samples and (ii) by adding three random nucleotide positions at the beginning of the sequence to improve cluster identification and to prevent phasing^38^. Despite our efforts, we cannot entirely rule out NGS-induced bias, since sequence similarity is an inherent problem when analyzing identically designed barcodes^39^,^40^. We did not focus on errors introduced by NGS e.g. during bridge amplification, because they were reported to be outnumbered by the PCR-introduced errors^23^.

In summary, we have shown that various amplification steps during barcode retrieval introduce a level of systemic bias that can be minimized, although not fully excluded. Most critically, the bias cannot be predicted based on sequence structure, thus making a latter (bioinformatical) correction challenging, especially without any prior knowledge about the initially utilized barcodes. However, by introducing a novel BC32 construct, we could demonstrate that mutual distance between the barcodes in terms of sequence similarity is a major determinant on whether bioinformatical post-processing allows to associate the descendent barcodes to their original sequence. We also suggest protocol adaptions for barcode calling, in particular the use of restriction enzymes to provide better access to the barcode sequence in combination with a reduction of PCR cycles.

Ultimately, our data demonstrate that a curated barcode library is essential for providing barcode kits that fulfil higher quality standards with respect to sequence integrity and distinguishability. While the establishment of a curated library is a tedious process, several of such libraries of different sizes and with different quality criteria will be necessary to meet varying demands in distinct experimental settings. Our data also emphasizes the need for a rigorous analysis of any barcode library in terms of sensitivity, specificity and precision including replicates and calibration experiments. In addition, we strongly recommend to report all details about PCR protocols and NGS settings within any relevant publication to ensure reproducibility of the experiments.

## Additional Information

**How to cite this article**: Thielecke, L. *et al*. Limitations and challenges of genetic barcode quantification. *Sci. Rep.* **7**, 43249; doi: 10.1038/srep43249 (2017).

**Publisher's note:** Springer Nature remains neutral with regard to jurisdictional claims in published maps and institutional affiliations.

## Supplementary Material

Supplementary Material
