# Supplementary material for: Limitations and challenges of genetic barcode quantification
Source: Sci Rep. 2017 Mar 3;7:43249. doi: 10.1038/srep43249 (PMC5335698; doi:10.1038/srep43249)
Supplement: Supplementary Material [file srep43249-s1.pdf]

Limitations and challenges of genetic barcode quantification

Lars Thielecke, Tim Aranyossy, Andreas Dahl, Rajiv Tiwari, Ingo Roeder, Hartmut Geiger, Boris Fehse, Ingmar Glauche and Kerstin Cornils

SUPPLEMENTARY DATA

Figure S1

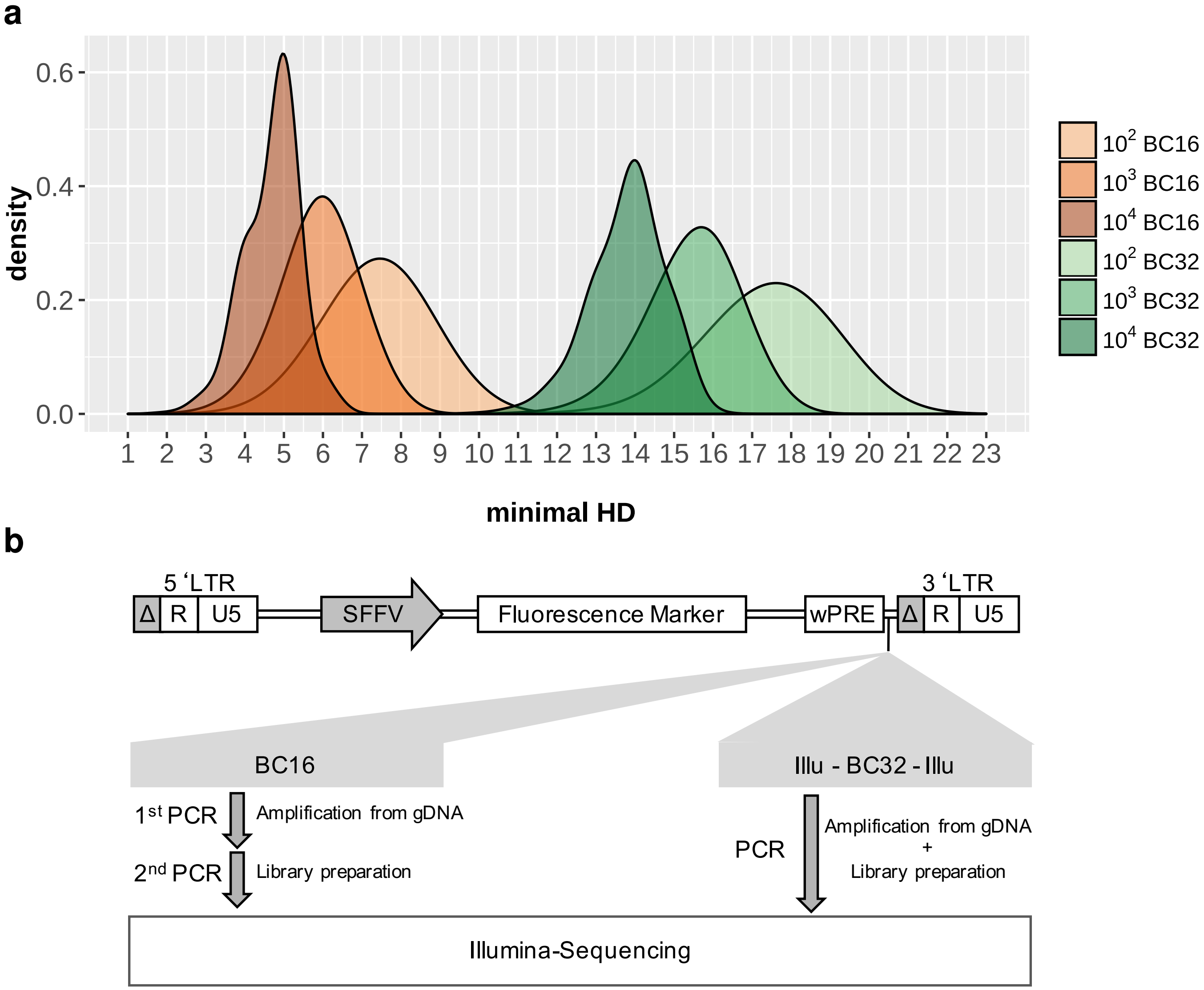

Fig. S1: (**a**) Simulated average Hamming distance distributions for a randomly picked sample of a 16 and 32 nucleotides long barcode and for three different initial cell numbers (averaged over 100 simulations). (**b**) Optimization strategy for the BC32 construct: additionally to the extension from 16 to 32 wobble bases, the construct was equipped with adaptors for Illumina-sequencing. These modules allow the amplification and NGS-library preparation in one PCR step and with less cycles.

Figure S2

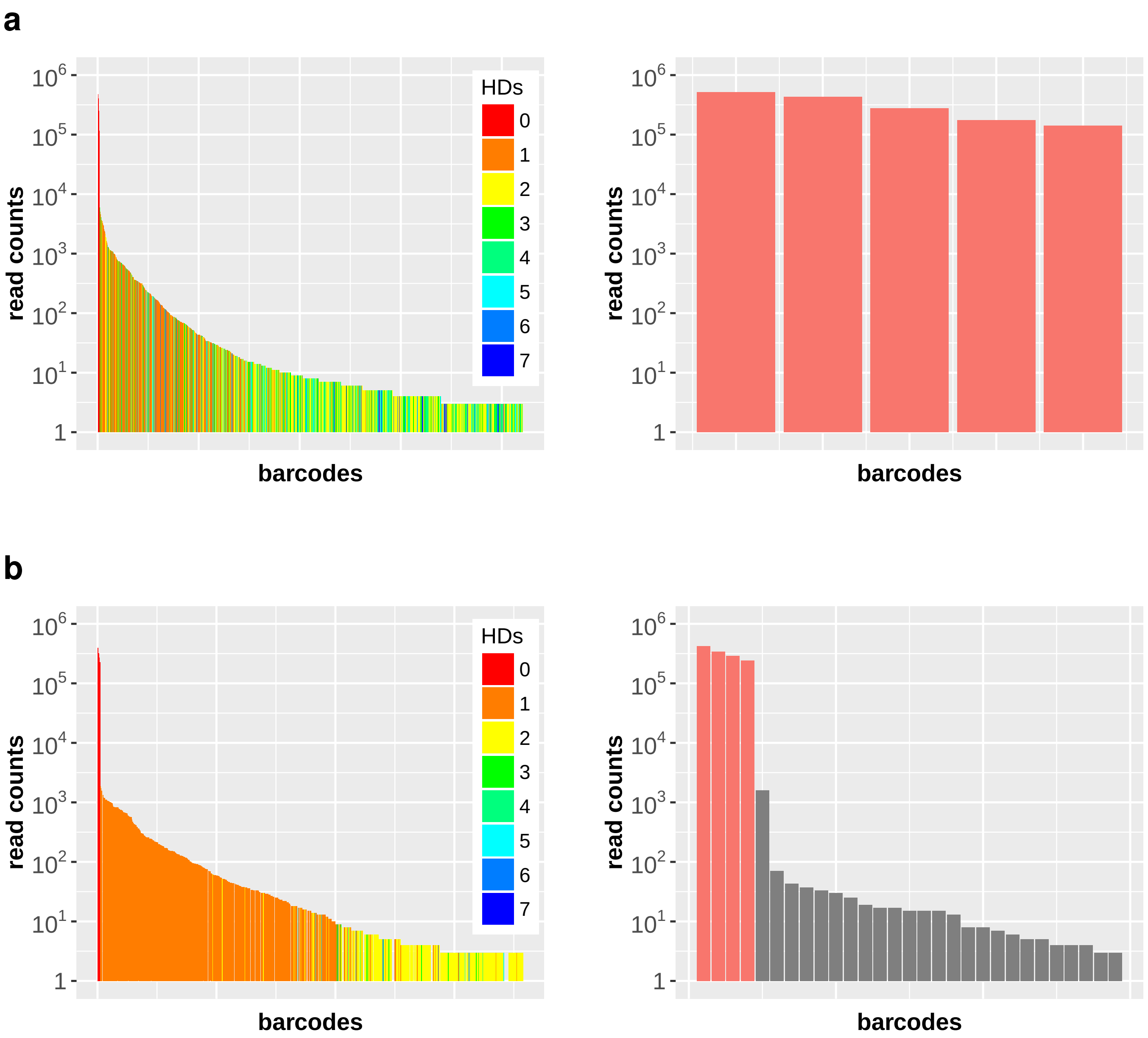

Fig. S2: Results of the error-correction method for one exemplary (**a**) BC16 and (**b**) BC32 miniBulk. The remaining barcodes (shaded in grey) can not be reliably assigned to any of the original barcodes and are most likely contaminations (HD > 16).

Figure S3

a

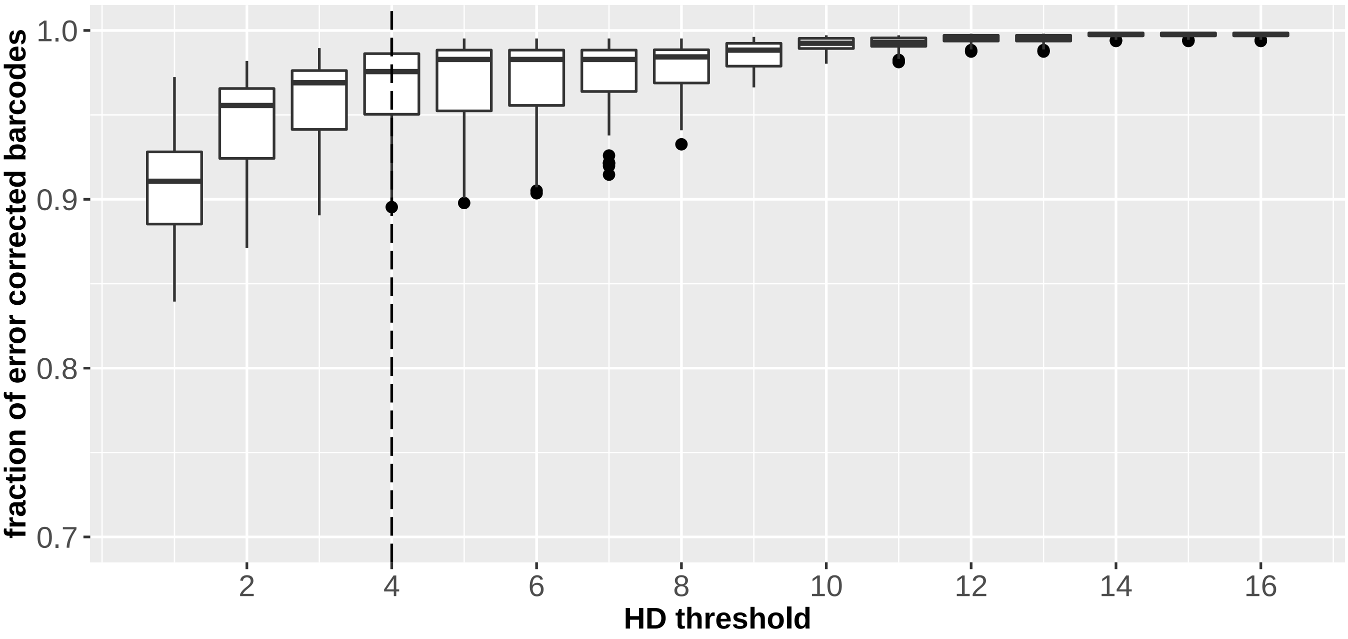

b

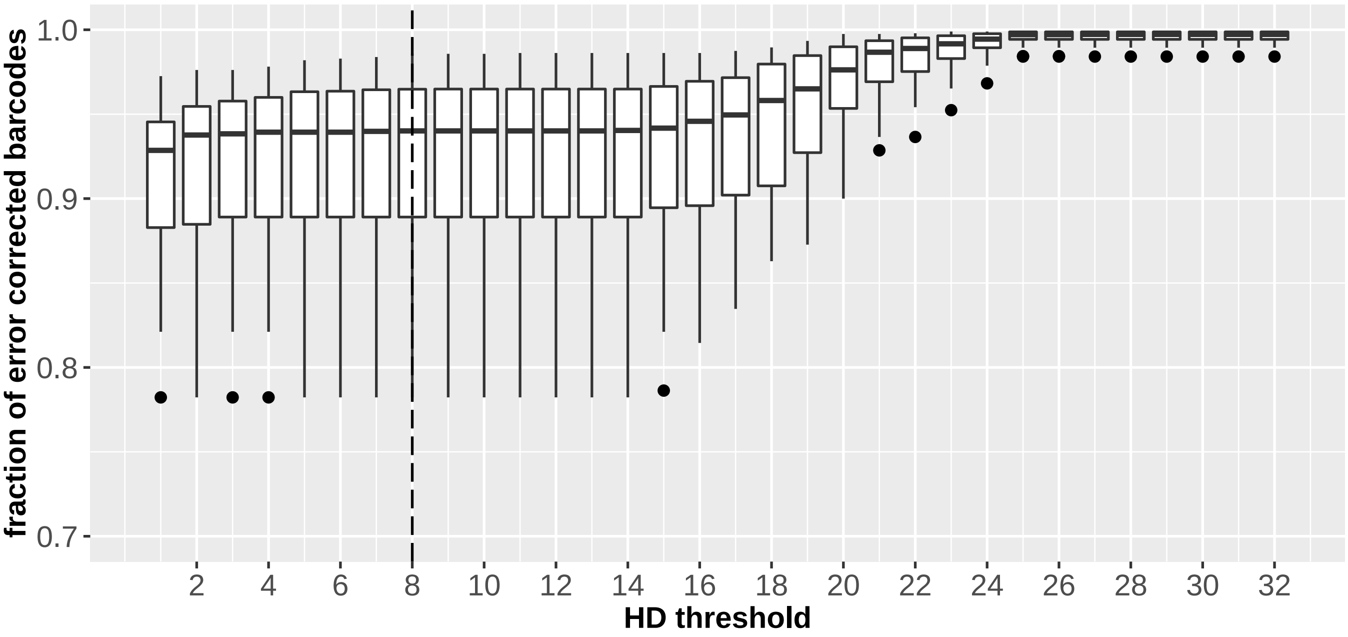

Fig. S3: Sensitivity analysis of the effect of the chosen Hamming distance (HD) threshold for the error correction model. The dashed line indicates the chosen upper Hamming distance threshold for the definition of similarity during error correction for both barcode constructs.

Figure S4

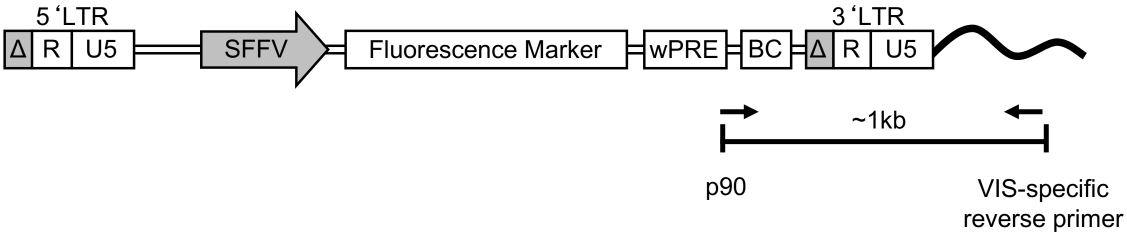

Clone	Genomic location	Reverse primer	Length of obtained fragment (bp)
BC16-B	Chr. 11q13.2	7 16BCw-1µL Kl.7	979
BC16-C1	Chr. 9q32	8 16BCw-1µL Kl.8	973
BC16-C2	Chr. 5q35.3	16BC-KlonC2-Chr	948
BC16-D	Chr. 21q22.3	9a 16BCw-1µL Kl.4	931
BC16-E	Chr. 9q34.3	10 16BCw-1µL Kl.10	930
BC32-A	Chr. 8q22.1	7 32BCw-5µL Kl.3	1088
BC32-G	Chr. 10q24.4	11a 32BCw-5µL Kl.9	1082
BC32-C	Chr. 17q25.3	9 32BCw-5µL Kl.5	1056
BC32-E	Chr. 19q13.42	10 32BCw-5µL Kl.7	1079

Fig. S4 Viral integration site analysis and generation of 1kb fragments. The viral integration site (IS) was identified via LM-PCR (26). To generate PCR fragments with approximately 1kb in length, we used a forward-primer located on the provirus (p90) and a IS-specific reverse primer. The obtained PCR-product contained the respective barcode-sequence of each clone

Figure S5

a

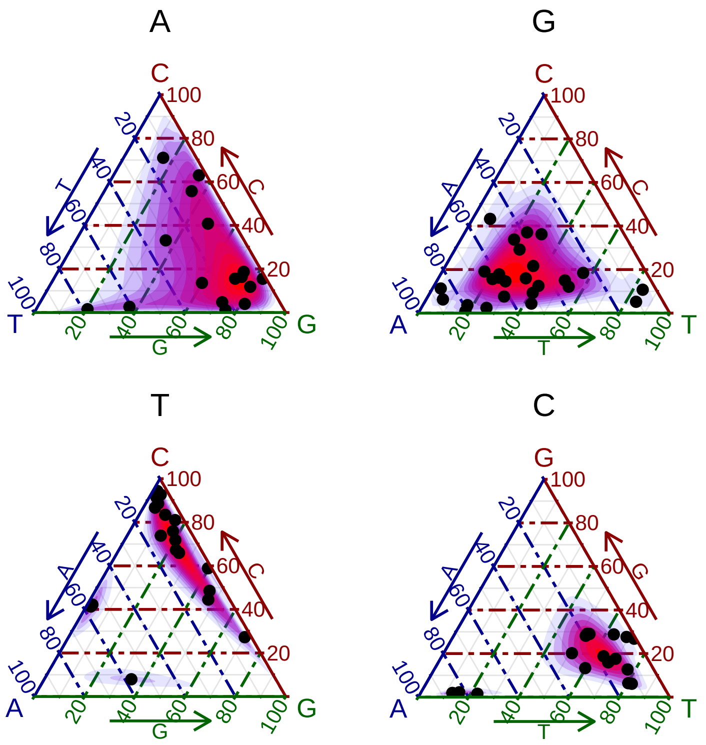

b

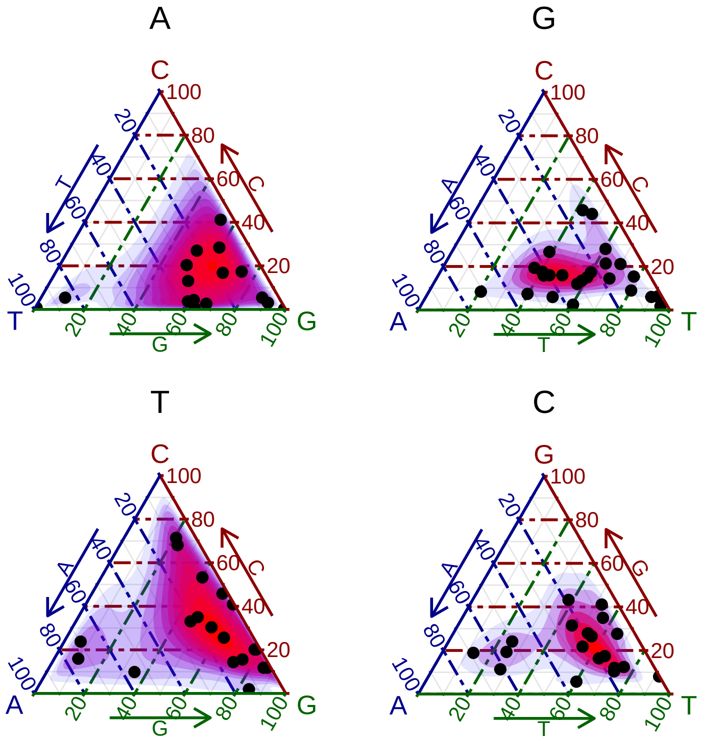

c

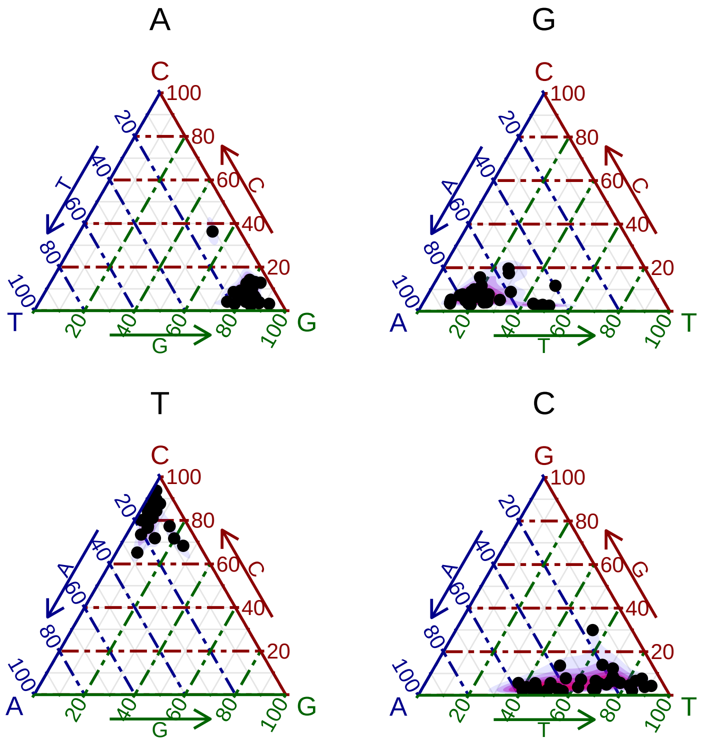

Fig. S5: Detailed inspection of mutation patterns on the level of single nucleotide substitutions for only the most similar descendent barcodes (with a HD = 1 and HD = 2 to the original barcodes) are displayed as ternary plots. Shown are the results for the BC16 construct (**a**), for the BC16 Q5 polymerase approach (**b**), and for the BC32 construct (**c**).

Figure S6

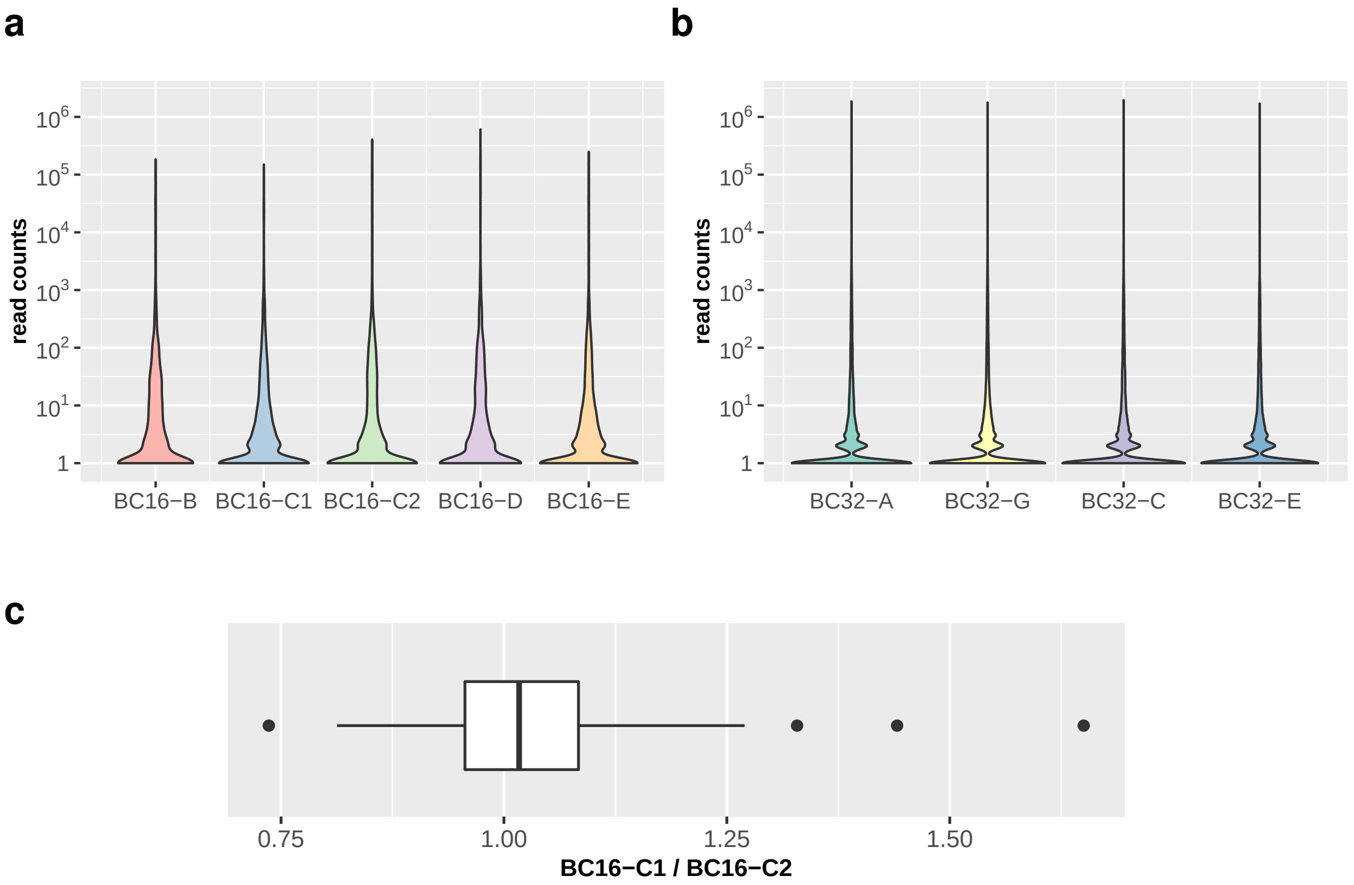

Fig. S6: Read count distribution of all identified descendent BCs for the (**a**) BC16 and (**b**) BC32 construct. (**c**) Read count ratio of the double integrated BC16-C1 and BC16-C2 BCs for all available miniBulks is depicted as boxplot.

Figure S7

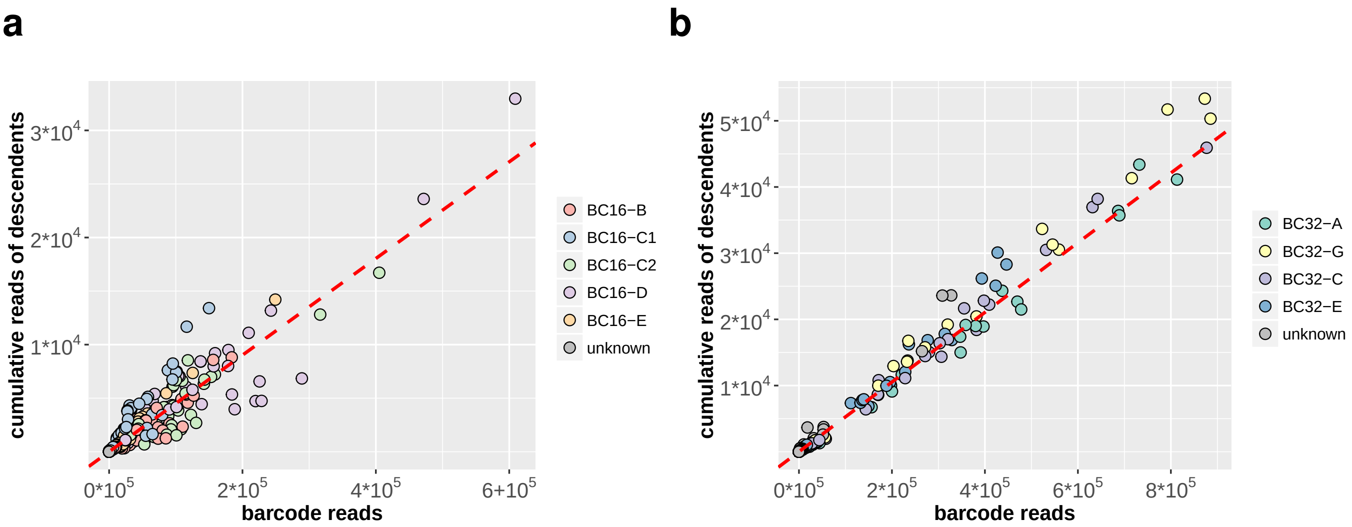

Fig. S7: The ratio of read counts obtained from the original barcodes vs their related descendent barcodes, according to the chosen thresholds of (**a**) HD = 4 for the BC16 and (**b**) HD = 8 for the BC32 construct.

Figure S8

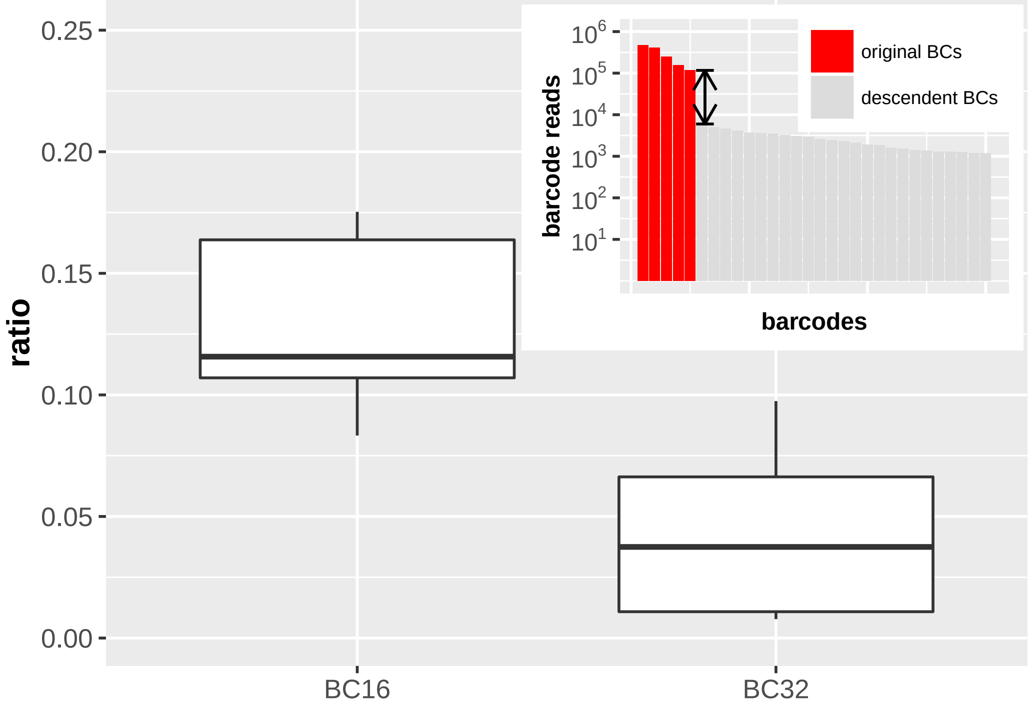

Fig. S8: The distribution of the relative read count ratio between the smallest original barcode and the biggest descendent barcode (BC$_{descendent}$/BC$_{original}$) for both of the studied barcode constructs is visualized as boxplots.

Figure S9

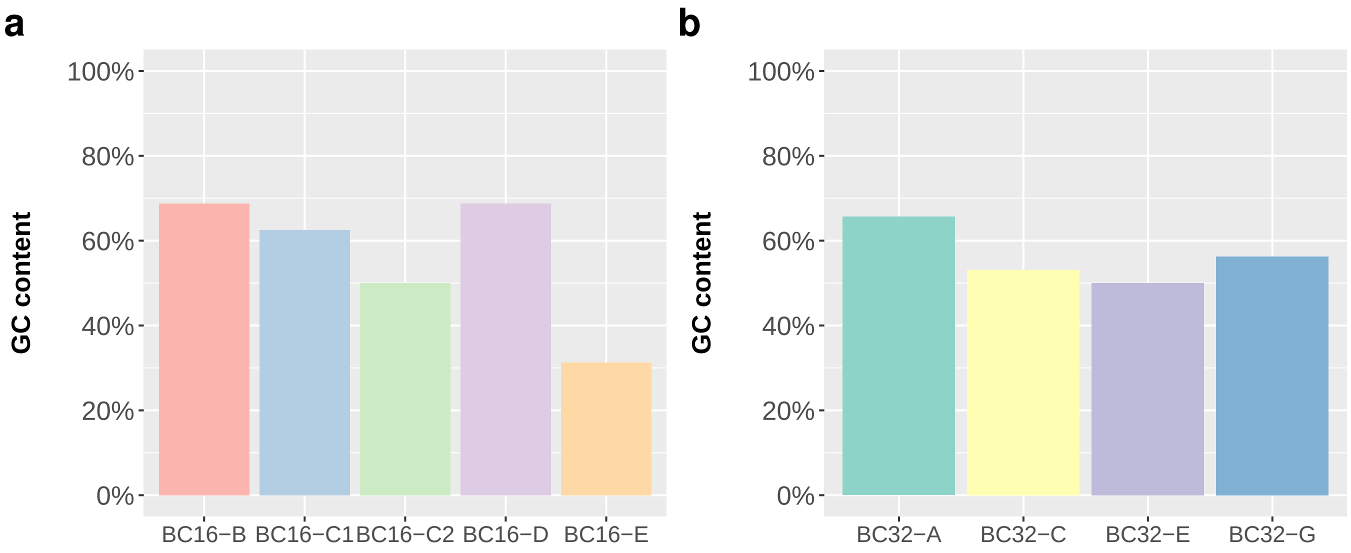

Fig. S9: GC content within the barcode sequences of the chosen (**a**) BC16 and (**b**) BC32 barcodes.

Figure S10

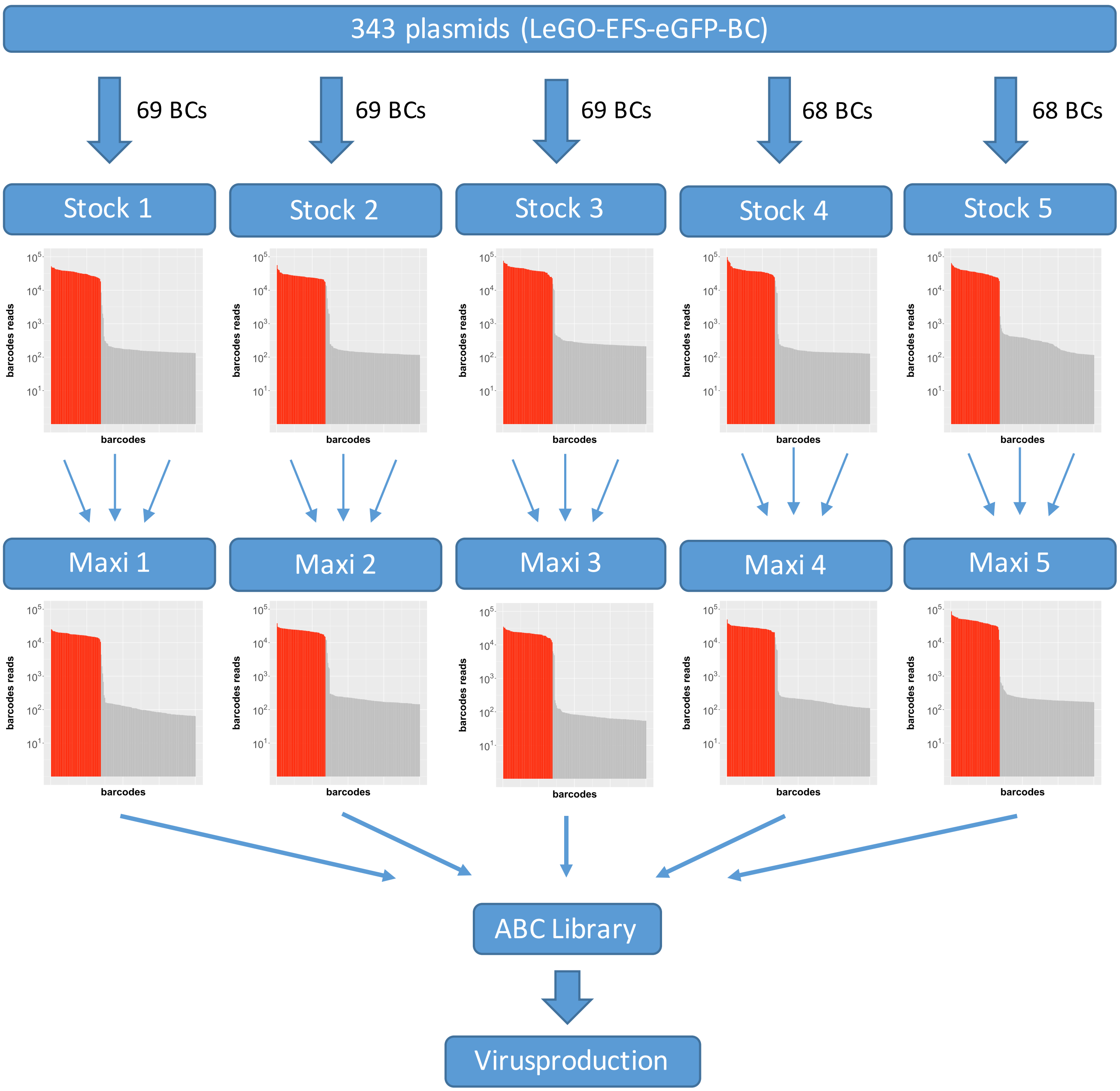

Fig. S10: 343 barcode equipped plasmids were selected and combined into five stocks, containing 68 or 69 different barcodes. After transformation and plasmid preparation, the five maxi-preparations were merged to generate the final annotated barcode library. As a quality control of the different steps, NGS of the selected plasmids for all stocks and the subsequently generated maxi preparations was performed.

Table S1

Function	Name	Sequence (5'->3')
**Barcode Oligos**		
**Generation of BC16**	Barcode-GFP-FW	CTAGAATCTANNACTNNCGANNCTTNNCGANNCTTNNGGANNCTANNGATCT
	Barcode-GFP-RV	TCGAGAGATCNNTAGNNTCCNNAAGNNTCGNNAAGNNTCGNNAGTNNTAGAT
**Generation of BC32**	Poly-GFP-barcode	ggtgCaTCTAGAACACTCTTTCCCTACACGACGCTCTTCCGATCTNNNACT<br>NNCGANNCTTNNCGANNCTTNNGGANNCTANNACTNNCGANNCTTNNCGAN<br>NCTTNNGGANNCTANNACTNNCGANNCTCGAGGTGCACTATG
	32BC-Poly-rev	CATAGTGCACCTCGAG
**PCR and Sequencing primers**		
**Barcode identification**	BC-PCR-FW	CATCGATACCGTCGACCTC
	BC-PCR-RV_neu	GCTAAGATCTACAGTCTCGAGAGATC
	BC-PCR-Seq	ACAGCAGCTACCAATGCTGA
**BC16 barcode retrieval**	BC-PCR-FW	CATCGATACCGTCGACCTC
	BC-PCR-RV_neu	GCTAAGATCTACAGTCTCGAGAGATC
	Ill2_Tail-complete	GTGACTGGAGTTCAGACGTGTGCTCTTCCGATCTCATCGATACCGTCGACC
	Ill1-Tail12	ACACTCTTTCCCTACACGACGCTCTTCCGATCTTCTCGAGAGATC
**BC32 barcode retrieval**	MPLX-primer	CAAGCAGAAGACGGCATACGAGATxxxxxxGTGACTGGAGTTC
	p43	GTGACTGGAGTTCAGACGTGTGCTCTTCCGATCT
	DUAL-Index-primer	AATGATACGGCGACCACCGAGATCTACACxxxxxxxxACACTCTTTCCCTA<br>CACGACGCTCTTCCGATCxT
**LM-PCR (5'LTR)**		
**Identification of second Integration in clone BC16-C**	LV-5-LTR-1	[biotin]-GGCCCTGGTGTGTAGTTCTG
	LV-5-LTR-2	CCTTCTCTTGCTCAACTGGT
	LV-5-LTR-3	AGCACCATCCAAAGGTCAGT
	LV-5-LTR-Seq	CTGCCAATCAGGGAAGTAGC
	OC1	GACCCCGGGAGATCTGAATTC
	OC2	AGTGGCACAGCAGTTAGG
**digital droplet PCR**		
**IS-specific ddPCR**	LV-Seq	CCCGTCTGTTGTGTGACTCT
	Lenti-LTR-BHQ	FAM-CCCTCAGACCCTTTTAGTCAGTGTGG-BHQ1
	7 16BCw-1µL Kl.7	TGGATTCCAATTGCTGTAGAGA
	8 16BCw-1µL Kl.8	GGTGCTGTGTGGCTTCTCTT
	16BC-KlonC2-Chr	TGCAGTTGTGAGTTAATATTGCA
	9a 16BCw-1µL Kl.4	TCACCAAGCACGTGTTATTTTC
	10 16BCw-1µL Kl.10	TTCTCGGTCATTCTCGATCC
	7 32BCw-5µL Kl.3	CATACCATACAGTTCACGCATTT
	11a 32BCw-5µL Kl.9	TTGCAGTTGAGTGAGCATGA
	9 32BCw-5µL Kl.5	GGCATGTGGCCATAGTCAGT
	10 32BCw-5µL Kl.7	AATTCCCAACTTGAAAGTAGCTC
**BC-specific ddPCR**	16BC-dPCR-FW	TGCCTGGCTAGAAGCACAAGA
	16BC-dPCR-P	FAM-TTTTCCAGTCACACCTCAGGTACCCAGC-BHQ1
	16BC-KlonB	CCCTAAGCGTCGGGAAGAG
	16BC-KlonC	CAAGGGTCGCGAAGTATCGT
	16BC-KlonC2	TCCCAAAGTGTCGCCAAGTAT
	16BC-KlonD	GATCGAGAAGGGTCGCAAGTAC
	16BC-KlonE	AAGATTCGTGAAGATTCGGTAGTGT
	32-dPCR-FW	CGAGAGATCGGAAGAGCACAA
	32-dPCR-P	FAM-CCCTACACGACGCTCTTCCGA-BHQ1
	32BC-KlonA-RV	CGGCAAGCCTCGGAAGT
	32BC-KlonG-RV	CAAGTGTTAGTGTCCCCAAGCC
	32BC-KlonC-RV	GTAGAATCCGCAAGGGTCGATA
	32BC-KlonE-RV	TCCCGAAGCCTCGCTAAGTA
**VCN ddPCR**	FP-dPCR-FW	CAGGAGCGCACCATCTTCTT
	FP-dPCR-BHQ	FAM-CTACAAGACCCGCGCCGAGGTGA-BHQ1
	FP-dPCR-RV	AGGGTGTCGCCCTCGAAC
**Housekeeping**	mEpo-fw	GCAGGCGGGGTCGCTACTC
	mEpo-probe	HEX-TTCTGAGGCGCCACTTTTGCAAGACC-BHQ1
	mEpo-rv	CGCCTGTGCAGATCCGATAA
**1kb fragments**		
	p90	GGACGTCCTTCTGCTACGT
	7 16BCw-1µL Kl.7	TGGATTCCAATTGCTGTAGAGA
	8 16BCw-1µL Kl.8	GGTGCTGTGTGGCTTCTCTT
	16BC-KlonC2-Chr	TGCAGTTGTGAGTTAATATTGCA
	9a 16BCw-1µL Kl.4	TCACCAAGCACGTGTTATTTTC
	10 16BCw-1µL Kl.10	TTCTCGGTCATTCTCGATCC
	7 32BCw-5µL Kl.3	CATACCATACAGTTCACGCATTT
	11a 32BCw-5µL Kl.9	TTGCAGTTGAGTGAGCATGA
	9 32BCw-5µL Kl.5	GGCATGTGGCCATAGTCAGT
	10 32BCw-5µL Kl.7	AATTCCCAACTTGAAAGTAGCTC

Table S2

ACTATCGAGCCTTGTCGATACTTCCGGATTCTACAACTGCCGACACTTCTCGACTCTTTCGGAGTCTATCACTATCGATC
ACTCTCGAAACTTATCGACCCTTGTGGACTCTACAACTACCGATACTTACCGAACCTTGCGGAGACTATCACTCGCGATT
ACTGGCGATTCTTGACGAACCTTTCGGATGCTACTACTCCCGACTCTTGACGACCCTTCGGGAATCTATCACTTTCGAAC
ACTCCCGAGTCTTTACGAATCTTCCGGATCCTACGACTCGCGATGCTTAACGATACTTCGGGACTCTATGACTTTCGAAT
ACTTTCGACGCTTCTCGAATCTTCCGGATGCTACAACTGCCGAACCTTGGCGAGCCTTCGGGAGGCTAGTACTGGCGACA
ACTTTCGAGACTTGTCGAACCTTAGGGACACTAACACTGCCGAGTCTTGTCGACACTTTCGGACACTACTACTACCGAAG
ACTTGCGAATCTTTCCGATCCTTTCGGAGTCTATTACTCGCGATGCTTACCGATTCTTATGGACTCTATTACTTTCGATT
ACTCCCGATTCTTAGCGATACTTATGGACACTAGAACTCCCGAAGCTTAGCGATCCTTCAGGATTCTACGACTCCCGACT
ACTGCCGAATCTTAACGATACTTTCGGAACCTATCACTTGCGATCCTTCGCGATACTTCGGGATGCTACAACTGCCGACT
ACTACCGATTCTTTGCGATGCTTTCGGACACTATGACTTACGATGCTTTGCGACGCTTCAGGAGGCTATTACTATCGATT
ACTTACGAATCTTTCCGACACTTCCGGAACCTAGGACTGACGACTCTTTCCGAAGCTTCAGGAATCTATAACTTGCGACT
ACTGCCGATGCTTCTCGATCCTTTCGGAACCTATTACTTCCGATCCTTTGCGACACTTTTGGAAACTATCACTAGCGAGT
ACTCACGACGCTTGACGAATCTTCCGGAACCTAGTACTGACGATTCTTCACGATACTTAAGGATACTAGTACTCCCGATT
ACTTCCGACGCTTGACGATTCTTTCGGACGCTAATACTGTCGACCCTTCTCGACTCTTGTGGATGCTAGTACTGCCGAAC
ACTCGCGAACCTTTGCGATGCTTCCGGAAACTAGGACTGTCGAACCTTACCGAGCCTTAAGGAACCTATTACTGGCGACA
ACTGGCGATGCTTCACGAACCTTGTGGATGCTACAACTAGCGATTCTTTGCGATTCTTAGGGAATCTATAACTTCCGACC
ACTCTCGATCCTTTACGATGCTTGGGGAGCCTATAACTCGCGAGGCTTGTCGACGCTTATGGAGGCTATGACTTGCGAAT
ACTACCGAACCTTTGCGATGCTTTAGGACGCTATCACTGGCGACACTTACCGATCCTTCGGGAGTCTATCACTCTCGATT
ACTATCGAGGCTTCGCGACACTTTGGGACACTAGCACTAGCGATCCTTGACGAACCTTTTGGAGCCTACCACTCACGAGC
ACTACCGATCCTTTTCGATCCTTTTGGAAACTATTACTGTCGAATCTTGTCGAGCCTTCCGGATTCTATCACTCTCGACC
ACTGACGACGCTTTTCGACGCTTATGGAATCTACGACTGACGAGCCTTTACGATCCTTCAGGAAGCTAGCACTTGCGACG
ACTTTCGAACCTTATCGATTCTTCGGGATTCTATTACTCTCGATTCTTTGCGAACCTTTAGGAGCCTATGACTATCGAGA
ACTCGCGATGCTTAGCGATGCTTTCGGAAACTACGACTCACGAATCTTAGCGAAGCTTTCGGAATCTATGACTGGCGAAT
ACTTACGAAGCTTTACGATACTTTCGGAACCTATCACTCGCGATCCTTTTCGAGCCTTAGGGACCCTATAACTATCGATG
ACTCCCGACACTTCTCGACCCTTATGGAGACTAGTACTATCGATGCTTGGCGACCCTTGCGGAAACTATTACTCGCGAAT
ACTTGCGATGCTTTCCGACACTTTTGGATGCTAAGACTGTCGACGCTTTTCGAGACTTCTGGATGCTAACACTGGCGACT
ACTTACGAGCCTTACCGAGCCTTGTGGAATCTAAAACTAACGACACTTTCCGAAACTTTCGGATACTAAAACTTCCGAAT
ACTATCGAGCCTTGTCGAGACTTCGGGAAGCTACCACTGCCGAATCTTCCCGACTCTTATGGATGCTATCACTCTCGACG
ACTGTCGAAGCTTGCCGATACTTCGGGAGCCTATGACTTTCGAACCTTCCCGATACTTGCGGATCCTATTACTGGCGACG
ACTACCGAAACTTAACGAGGCTTACGGACACTACGACTTCCGAATCTTTCCGAGCCTTCCGGACTCTATAACTGCCGAGT
ACTCGCGAGACTTGACGATGCTTGCGGACTCTAGTACTCACGATTCTTGGCGATACTTTTGGAGTCTACGACTAACGATT
ACTGTCGACTCTTGGCGAGTCTTCAGGAAACTACTACTTTCGACACTTGACGACCCTTTTGGATCCTACCACTTGCGAGA
ACTTGCGATCCTTACCGAATCTTGAGGATACTACTACTCTCGATCCTTTGCGAGTCTTCAGGAGTCTATTACTTCCGACC
ACTATCGAGGCTTTTCGACGCTTTCGGAGTCTATAACTACCGAAGCTTCCCGAGACTTGGGGATACTAGTACTGCCGAGC
ACTTTCGACGCTTCCCGAATCTTCAGGATTCTACAACTTACGATTCTTGCCGATTCTTTTGGAGTCTAACACTCCCGATG
ACTTCCGAAACTTCACGACCCTTGTGGATCCTACAACTTACGATGCTTGCCGATACTTAAGGACTCTATTACTGTCGACC
ACTGACGACCCTTCTCGACTCTTTAGGATCCTACCACTTACGAAGCTTTGCGAACCTTCCGGAGTCTACTACTGCCGATC
ACTCACGATACTTTGCGAGCCTTCTGGACCCTAATACTCTCGATCCTTTTCGACACTTGTGGAATCTATTACTCACGACC
ACTTACGACGCTTGCCGATACTTTGGGACGCTATGACTTGCGACCCTTGGCGAACCTTTTGGAGTCTACGACTTGCGAAT
ACTGCCGATTCTTCCCGACCCTTAGGGATACTATCACTTACGATACTTACCGACTCTTATGGAGACTATCACTTCCGACT
ACTTTCGATACTTCTCGAACCTTTAGGAGACTAGGACTCACGATTCTTGGCGATTCTTGGGGACACTATTACTATCGACC
ACTTCCGAAACTTTACGATGCTTTCGGATCCTATAACTGGCGATTCTTTTCGAGTCTTGTGGACACTACCACTGGCGAGC
ACTTTCGAGCCTTGTCGACTCTTACGGATTCTACCACTGGCGATTCTTTTCGATCCTTCTGGATCCTATGACTCACGAGG
ACTTTCGAAACTTTACGAACCTTGAGGACTCTAGTACTACCGACTCTTCCCGATTCTTCAGGACACTACGACTCTCGAGA
ACTCCCGAGCCTTTACGATGCTTTCGGAATCTAGCACTAGCGAATCTTATCGATTCTTTAGGAGGCTATAACTTTCGACC
ACTATCGAGCCTTGCCGACTCTTCTGGACCCTATGACTCACGAGCCTTCTCGACGCTTAGGGATACTAACACTTCCGAGC
ACTCTCGACACTTTTCGATCCTTCTGGACTCTACAACTGACGATACTTCCCGACTCTTGTGGACCCTAACACTCTCGACG
ACTGTCGAAACTTTTCGATCCTTTCGGAGCCTAAAACTAACGATCCTTGTCGACGCTTCTGGAGCCTAGTACTTGCGATG
ACTCACGATCCTTCACGAGGCTTCTGGACTCTACTACTGTCGATACTTCACGAACCTTCCGGACCCTATAACTTCCGAGG
ACTAGCGAAGCTTATCGAGGCTTGTGGATACTATAACTAGCGATGCTTGGCGATTCTTTCGGATCCTATCACTTACGAGC
ACTCGCGAAACTTTTCGAATCTTGCGGAGGCTAGCACTCGCGACCCTTCACGACCCTTACGGATCCTATAACTATCGATC
ACTGTCGAATCTTCCCGAAGCTTAGGGATTCTACTACTACCGAAGCTTTTCGATACTTCTGGATACTAGTACTTCCGAAT
ACTATCGAGACTTACCGACCCTTCGGGAGTCTAGCACTGACGACACTTGTCGACCCTTGGGGATCCTATTACTTACGACG
ACTTCCGACGCTTTTCGAGCCTTCCGGAAACTAACACTCTCGAATCTTCACGAGGCTTGCGGACACTAAGACTCTCGAGA
ACTGTCGAAACTTGGCGATGCTTAGGGACACTATGACTGGCGAGCCTTAGCGAGGCTTCCGGAGACTAAGACTACCGACT
ACTTCCGATCCTTTACGACGCTTTGGGAGGCTACCACTCGCGACCCTTCTCGACTCTTACGGAGCCTAAAACTGTCGACG
ACTCTCGACACTTATCGAGCCTTCGGGATCCTAAAACTTTCGAATCTTGCCGATGCTTTCGGATACTATTACTCACGATT
ACTACCGAGCCTTGACGACACTTAAGGACGCTACTACTAGCGATACTTACCGAGGCTTCTGGATACTAACACTTTCGACA
ACTACCGAGACTTCGCGAAACTTCTGGATCCTATCACTCTCGAACCTTACCGAAACTTGTGGAGGCTATCACTTCCGATT

ACTGGCGACGCTTGTCGATTCTTTCGGACGCTATAACTCGCGATCCTTTTCGAGGCTTGAGGACTCTAACACTCGCGACG
ACTCCCGACACTTTCCGACACTTGCGGATACTACGACTAACGAAGCTTTCCGAATCTTACGGATCCTATCACTAACGACG
ACTTTCGAACCTTTGCGAGTCTTTCGGACGCTAAAACTCCCGATACTTTCCGAATCTTGTGGAAGCTAGCACTGTCGAAT
ACTCACGAGACTTCTCGACCCTTGAGGACTCTACCACTTACGATGCTTCGCGAACCTTAAGGAATCTATGACTTTCGAGT
ACTCACGAGACTTTCCGACGCTTACGGATTCTATGACTTACGACCCTTAACGAATCTTTCGGAACCTAGCACTGACGACT
ACTGCCGATGCTTGTCGATGCTTGTGGAGCCTATGACTATCGAGGCTTCGCGAATCTTATGGAAGCTACCACTTTCGATT
ACTCACGATGCTTTCCGAAACTTCGGGAATCTATAACTATCGATTCTTATCGATACTTCCGGATTCTACAACTTGCGACC
ACTAGCGACACTTGCCGATACTTAAGGATGCTATAACTCACGACACTTGTCGAGACTTATGGATGCTAGCACTGGCGATC
ACTCCCGACTCTTGCCGATTCTTTCGGACACTATAACTTTCGACTCTTACCGAATCTTACGGATTCTAGCACTACCGACC
ACTGCCGACTCTTCTCGATACTTCTGGATACTAGAACTGCCGACCCTTAACGACACTTTCGGATGCTAGCACTTCCGAAT
ACTTCCGACTCTTCGCGACACTTTGGGATTCTATGACTCACGAAGCTTCTCGAATCTTCTGGACGCTAGGACTTTCGACT
ACTGCCGATTCTTGGCGACTCTTGCGGAACCTATCACTCGCGAATCTTTTCGATCCTTTTGGACACTATCACTTGCGATC
ACTTCCGAATCTTCTCGACCCTTTTGGAGCCTACAACTCCCGACGCTTCTCGACTCTTCTGGATGCTACTACTCGCGATG
ACTCGCGAGACTTTACGACGCTTTTGGAGGCTATTACTTTCGAGCCTTCCCGACGCTTTTGGAGGCTAGCACTCGCGACC
ACTCCCGAATCTTGTCGAAACTTCAGGATTCTATGACTGGCGACACTTACCGATGCTTTGGGACTCTAACACTCACGAAC
ACTCGCGACCCTTTTCGATACTTGGGGATCCTACTACTCACGATACTTTTCGATGCTTCTGGAATCTACCACTTGCGAAT
ACTCCCGACACTTTTCGAGTCTTGTGGAATCTAGCACTAGCGAATCTTTCCGAACCTTGCGGACTCTACAACTCACGATA
ACTTACGACGCTTACCGAATCTTCTGGACTCTACGACTGCCGACACTTCACGACCCTTGAGGATCCTATAACTTGCGATG
ACTCCCGAAACTTGCCGATTCTTCTGGAGTCTACCACTTGCGACACTTCACGATACTTGAGGACACTAAAACTACCGATA
ACTCCCGATTCTTTGCGAAACTTGTGGATGCTAATACTAACGAGACTTTCCGACCCTTGGGGATACTACAACTTTCGAAC
ACTCTCGACCCTTTACGAGACTTGCGGACGCTATAACTATCGATCCTTGACGATGCTTCTGGATTCTACAACTTGCGAAT
ACTATCGACTCTTATCGAACCTTCGGGAGGCTACTACTCGCGACACTTTTCGATTCTTCTGGATCCTATAACTTTCGATA
ACTGGCGAGCCTTGACGATTCTTGTGGAAGCTAATACTTCCGACCCTTCACGACGCTTTAGGATACTAGTACTCACGATA
ACTTTCGACTCTTCACGAAGCTTCAGGATGCTAACACTTACGACTCTTTTCGAAACTTTGGGATGCTATCACTTGCGAGC
ACTCACGACTCTTTGCGATACTTTTGGAGTCTACAACTATCGAATCTTGACGACCCTTGCGGATGCTACGACTCCCGATG
ACTCGCGACGCTTTCCGATTCTTGCGGAATCTAACACTGGCGATCCTTACCGAAACTTTGGGACGCTATCACTTCCGAAC
ACTGTCGAGACTTTGCGAACCTTTTGGAGGCTAATACTTACGATTCTTGCCGATGCTTTCGGACCCTATCACTTCCGATT
ACTAACGATTCTTGACGAATCTTATGGAACCTAAAACTCTCGACTCTTCACGACCCTTGAGGATCCTATGACTCCCGAAG
ACTGCCGACACTTTTCGACCCTTACGGACACTAGTACTTACGACGCTTAACGAATCTTTAGGAGCCTACTACTTTCGAAT
ACTTCCGATACTTTGCGACTCTTATGGAAACTACTACTGGCGAGCCTTTTCGAGTCTTGTGGATACTATTACTCTCGATC
ACTTCCGAGGCTTTCCGACGCTTCAGGACGCTAATACTGCCGAGCCTTGTCGAGACTTTAGGATCCTACCACTGACGATC
ACTCCCGAGACTTCGCGAGACTTACGGACCCTAACACTTACGAGCCTTTTCGAGGCTTCTGGACCCTATAACTGTCGATA
ACTCTCGATACTTTACGAGTCTTTCGGAATCTAACACTTGCGATGCTTATCGAAGCTTTCGGATTCTATTACTTTCGACT
ACTTTCGATCCTTCACGACCCTTGCGGATACTAGAACTTTCGACACTTCCCGATCCTTGGGGATCCTAGAACTCTCGATT
ACTGTCGATACTTAACGAGCCTTCAGGATTCTACGACTAACGATGCTTATCGACACTTGCGGACACTATGACTCCCGAAC
ACTTCCGAAGCTTTCCGAATCTTCAGGAGACTAATACTAGCGATGCTTTCCGAGTCTTTAGGAATCTATCACTACCGACT
ACTGCCGATTCTTCCCGATGCTTTCGGAGGCTAATACTCCCGAGCCTTCGCGAGACTTTCGGATACTATCACTTTCGAGC
ACTTGCGAGCCTTGACGAAGCTTCGGGATGCTATGACTTTCGAAACTTTTCGATCCTTGCGGACACTACTACTTCCGATC
ACTTACGAGACTTCCCGACACTTCTGGATACTAGGACTGGCGAGACTTCTCGAAGCTTTGGGACTCTATGACTGTCGATC
ACTACCGACCCTTTACGAGGCTTATGGATCCTATAACTCGCGACCCTTAACGATGCTTCGGGACTCTACTACTCCCGACC
ACTCGCGATTCTTTGCGACGCTTCAGGACTCTAGCACTAACGATCCTTTCCGATTCTTCCGGATCCTACTACTTGCGAAC
ACTGTCGACACTTAGCGATCCTTCGGGACGCTATTACTACCGATTCTTTACGACTCTTTCGGACGCTACCACTGTCGAGC
ACTGACGAATCTTCACGAGTCTTACGGAACCTACTACTATCGAGTCTTAGCGAAACTTGTGGATACTACCACTGGCGATC
ACTATCGACACTTAGCGATTCTTAAGGACTCTATAACTCGCGATGCTTTGCGAGACTTAAGGATTCTATTACTCTCGATT
ACTTACGATCCTTGGCGAACCTTGAGGAGTCTAAAACTGGCGACTCTTTCCGACTCTTCTGGACCCTAATACTGTCGAGT
ACTTACGATCCTTACCGATACTTCGGGAATCTAAAACTAGCGAATCTTACCGAGGCTTATGGACTCTATGACTGCCGACA
ACTAACGATTCTTTCCGACGCTTTGGGAATCTAACACTGTCGACACTTGCCGATACTTTGGGATCCTATCACTGCCGAAA
ACTTACGACCCTTTTCGATTCTTATGGAACCTATTACTTTCGACGCTTCTCGATTCTTCAGGAAGCTACCACTCCCGAAC
ACTACCGAACCTTTTCGATCCTTTGGGAAGCTACCACTACCGATTCTTTACGAATCTTAGGGACGCTAAAACTACCGAGG
ACTTGCGAACCTTGTCGACTCTTGCGGAATCTAAAACTCTCGATACTTCACGAATCTTCCGGATCCTAGTACTCACGATT
ACTTGCGAAACTTTCCGAGCCTTATGGATCCTACGACTGCCGACGCTTACCGATGCTTGGGGAGGCTAGGACTGTCGACA
ACTCCCGACTCTTGACGATTCTTTTGGATTCTAACACTGTCGATGCTTATCGAGCCTTGTGGAACCTAACACTCTCGATA
ACTCACGAGCCTTAACGAGGCTTGGGGAAACTAGTACTACCGATACTTAGCGATTCTTTCGGACTCTACTACTTCCGACA
ACTCGCGAAACTTCGCGACGCTTACGGATTCTACTACTATCGAACCTTCCCGATGCTTGCGGACTCTACGACTTGCGAAT
ACTTTCGAGTCTTCCCGAACCTTCCGGAGTCTAGCACTCTCGATGCTTTGCGAGACTTCTGGACCCTAGCACTCACGAGT
ACTCTCGAATCTTAACGAGACTTTCGGACGCTAATACTGCCGATTCTTTTCGAGGCTTCGGGATGCTACTACTTACGACC
ACTGACGACCCTTGCCGAGCCTTGCGGAAACTATAACTGCCGAGACTTGGCGACCCTTGGGGAACCTAGAACTGTCGACG
ACTTTCGAATCTTAACGATTCTTGTGGACACTAGGACTTCCGACTCTTGACGAAACTTTCGGAACCTAATACTGTCGAGG
ACTTGCGAAGCTTTCCGACTCTTGCGGAGCCTAGTACTCACGAGGCTTTCCGAAGCTTCTGGAAGCTAAAACTCGCGAGT
ACTAGCGATACTTCGCGACCCTTTGGGAGTCTATTACTACCGAAGCTTGACGACGCTTAGGGACTCTATTACTTCCGACT
ACTCTCGAACCTTACCGACACTTTTGGACGCTATCACTTTCGACCCTTGTCGACACTTCAGGATCCTAGCACTCGCGAAC
ACTCGCGACTCTTCACGAGGCTTTAGGATTCTACCACTTTCGAGGCTTCGCGATTCTTGCGGAAGCTAGTACTCTCGACA

ACTACCGAGTCTTATCGATTCTTCAGGACTCTACTACTGCCGACCCTTGACGAGGCTTGAGGATACTAATACTTACGAGG
ACTCACGAGACTTAACGATCCTTGGGGACTCTATTACTTTCGAAGCTTCCCGAGTCTTGCGGATCCTATAACTCTCGAGA
ACTACCGATCCTTCGCGACACTTAGGGAATCTACTACTGGCGAGGCTTAACGATACTTCGGGACCCTAATACTACCGACG
ACTGTCGATTCTTCCCGACCCTTTTGGACTCTACAACTCACGAATCTTCGCGAGACTTGGGGAAACTATGACTTACGATC
ACTCTCGACTCTTATCGATTCTTGTGGATACTACTACTACCGATTCTTTACGACGCTTTAGGAGCCTAATACTCACGATC
ACTCCCGATGCTTCGCGACACTTTCGGAGGCTATCACTTCCGAAGCTTGACGACCCTTCAGGATCCTAGTACTGCCGACA
ACTAGCGAGTCTTATCGACCCTTACGGAATCTACTACTCGCGAAACTTGCCGACACTTATGGACGCTAGAACTAACGAAC
ACTTCCGATGCTTTGCGAAACTTAGGGAGTCTACCACTCCCGAGGCTTTCCGACACTTTTGGATTCTACTACTGCCGAAT
ACTGGCGATCCTTAGCGACACTTTTGGACTCTATCACTTGCGAGGCTTCTCGATTCTTCAGGAGCCTAGTACTGTCGATG
ACTCCCGACACTTCTCGACCCTTTCGGAATCTATGACTACCGACACTTCGCGAGCCTTAAGGACACTACTACTACCGATT
ACTCTCGAATCTTCACGAATCTTATGGATCCTACGACTAACGAAGCTTGTCGACACTTTTGGATCCTAAGACTTCCGATA
ACTGTCGATCCTTGTCGATGCTTGTGGACTCTACCACTTCCGAGGCTTGTCGAGACTTATGGACCCTATCACTTTCGAAT
ACTTCCGACTCTTTGCGAGACTTCGGGACTCTATGACTAACGAGACTTCGCGACACTTCGGGACCCTAAGACTCCCGATT
ACTCACGATTCTTCTCGATACTTGTGGAAGCTACCACTCGCGACACTTATCGATACTTGCGGAGCCTACGACTACCGATC
ACTTGCGATCCTTTCCGACACTTGGGGAGACTATTACTGGCGATTCTTATCGATTCTTTTGGAATCTAGTACTCGCGATT
ACTAGCGAACCTTGCCGAGCCTTAGGGAAGCTAACACTCCCGAGGCTTTGCGATACTTGCGGAGGCTATAACTGCCGATT
ACTGGCGAACCTTTTCGACCCTTTCGGACTCTAGCACTCCCGACGCTTAACGACACTTTTGGAATCTAACACTCCCGACC
ACTCGCGAAACTTCCCGAGCCTTCGGGAATCTACAACTATCGAGACTTAGCGACTCTTCCGGAGGCTAGAACTTACGATC
ACTGTCGATGCTTTGCGACCCTTCTGGATCCTAACACTCACGAATCTTTGCGATCCTTTCGGATTCTACGACTTCCGAAT
ACTCGCGAAACTTGCCGATCCTTTAGGAGGCTATTACTCACGATCCTTATCGAGCCTTTAGGAACCTAACACTTTCGAAC
ACTCTCGACCCTTCGCGACGCTTTTGGAGACTAAGACTGTCGACACTTCCCGAGTCTTCAGGATACTAACACTTTCGACG
ACTTGCGACGCTTATCGAAACTTTAGGACGCTATCACTTGCGATTCTTTACGACCCTTGGGGAACCTAGAACTTTCGAAC
ACTGACGAGCCTTACCGACCCTTCTGGAACCTACTACTTACGAATCTTTTCGATCCTTGTGGAACCTATCACTGGCGATG
ACTATCGATGCTTCTCGACGCTTGGGGAAGCTACCACTCTCGACTCTTCACGATTCTTGCGGATGCTAATACTTCCGATT
ACTCTCGATTCTTTTCGAAGCTTTTGGATTCTACTACTAGCGAGTCTTTGCGACTCTTATGGACTCTAATACTAACGAGG
ACTACCGAGCCTTCTCGATCCTTTAGGATGCTATTACTCGCGAGGCTTCACGACCCTTGCGGAAGCTAACACTTCCGATG
ACTTTCGATCCTTTTCGAAACTTAGGGAGACTAATACTAGCGAATCTTGGCGATCCTTAAGGACACTACCACTTGCGACG
ACTCACGACGCTTAACGACGCTTTTGGAGCCTACTACTAGCGAGGCTTCTCGATACTTCTGGACTCTAATACTCTCGACC
ACTGGCGATACTTTACGAACCTTACGGATGCTAATACTTCCGAGCCTTAGCGACTCTTTCGGACCCTAACACTCGCGATC
ACTAACGATCCTTTCCGACACTTCCGGACTCTAGCACTGGCGAGTCTTTGCGATTCTTTTGGAATCTATCACTCTCGAAT
ACTGACGAAGCTTAACGACGCTTACGGAACCTATTACTTGCGACTCTTATCGACACTTTTGGACCCTACCACTGCCGATA
ACTGGCGAATCTTAACGATGCTTGGGGAGCCTACAACTATCGATACTTCACGATGCTTTAGGAGTCTACCACTGCCGAGT
ACTCGCGAAACTTGGCGATGCTTGGGGAAGCTACCACTTTCGAAGCTTCTCGACCCTTAGGGACCCTAATACTGTCGAGC
ACTATCGACACTTAGCGACACTTTAGGAGGCTATGACTATCGATTCTTAGCGAAACTTCGGGATACTACCACTGCCGAAG
ACTGTCGAGGCTTATCGATACTTGCGGACCCTATAACTACCGACTCTTCCCGACCCTTATGGACTCTAATACTCTCGAGA
ACTTTCGAGCCTTCCCGAGTCTTTGGGATACTACTACTTGCGACGCTTATCGATTCTTCCGGATACTAGTACTAGCGATG
ACTTTCGACGCTTCTCGATCCTTTGGGATTCTAGCACTTCCGAACCTTTTCGACCCTTTAGGATACTAACACTATCGACC
ACTCGCGAGCCTTGGCGAGCCTTGTGGACACTATAACTCGCGATACTTCGCGATTCTTTTGGATTCTACTACTGTCGATC
ACTCCCGATGCTTTCCGACGCTTACGGAAGCTAGCACTCACGATCCTTGTCGAAGCTTGAGGACACTATTACTCTCGACG
ACTCCCGATGCTTATCGATACTTTTGGAAGCTAAAACTTCCGACCCTTGACGACCCTTTCGGATGCTATTACTCGCGACA
ACTGCCGACTCTTTTCGACACTTTGGGACGCTAATACTATCGACTCTTTGCGACGCTTGAGGATCCTAGGACTCACGATG
ACTGTCGATTCTTTGCGACACTTAGGGACGCTACTACTGCCGAGGCTTTGCGATGCTTGTGGAACCTACCACTCACGAGC
ACTAGCGACTCTTCACGATACTTTTGGAGACTACGACTTACGACCCTTCTCGAACCTTGCGGAGCCTACTACTCTCGAGA
ACTTGCGATCCTTCCCGATTCTTAGGGATACTACCACTACCGAACCTTAACGAGACTTGCGGATTCTATTACTGACGAGC
ACTGTCGATTCTTCTCGATTCTTCGGGAGTCTAGCACTGGCGAGCCTTCTCGAAACTTTTGGACGCTAAAACTCTCGAAT
ACTCCCGAGACTTGGCGAGCCTTTCGGAGTCTACTACTCACGAGACTTTCCGAGGCTTTCGGAACCTATAACTCGCGAAC
ACTCACGAACCTTATCGAACCTTTGGGAGTCTAGTACTCGCGAGCCTTGTCGACCCTTGAGGAGCCTAATACTGCCGATT
ACTGTCGATTCTTCTCGAAACTTACGGATCCTATTACTTGCGACACTTAACGAGCCTTATGGACCCTACGACTATCGACG
ACTTCCGATCCTTCTCGAACCTTTTGGATACTATAACTTGCGATCCTTATCGAATCTTCAGGAGTCTAACACTGCCGACC
ACTCGCGAGGCTTTGCGATCCTTGTGGACCCTACTACTTACGAATCTTCCCGAAACTTCCGGACTCTATCACTAACGAAA
ACTTTCGATACTTTTCGATGCTTCAGGAACCTACCACTAACGAATCTTGCCGACCCTTTCGGACTCTACCACTTCCGATT
ACTTTCGAGCCTTGTCGATGCTTCTGGAAACTATTACTCTCGAATCTTACCGACACTTTCGGAGACTAGGACTGTCGACG
ACTACCGAGGCTTAGCGACGCTTGAGGATGCTAACACTTCCGAGCCTTTTCGATGCTTCCGGAATCTAAGACTATCGATC
ACTCCCGACCCTTGCCGAATCTTTGGGATCCTAACACTGTCGATACTTTCCGATCCTTACGGACCCTACAACTTCCGATC
ACTTTCGAACCTTCCCGACGCTTTGGGAAGCTAACACTGCCGAAACTTGGCGATTCTTGTGGATACTATAACTAGCGAGG
ACTTCCGAAACTTGGCGAAACTTCCGGAGACTATAACTCACGACTCTTTTCGAATCTTGGGGACCCTAAGACTGGCGACG
ACTTTCGAACCTTTTCGACACTTGTGGAACCTATCACTCGCGATCCTTTCCGACGCTTCAGGAAGCTAACACTGACGATG
ACTCGCGACACTTAACGACACTTTAGGATCCTAAGACTGGCGATTCTTCTCGATGCTTTAGGAACCTATAACTGTCGATC
ACTAGCGATCCTTATCGAATCTTCCGGAAACTAGAACTTCCGATCCTTATCGACACTTGCGGACGCTACTACTCTCGACT
ACTTTCGATACTTCCCGACCCTTAAGGATCCTACGACTTTCGAAACTTACCGATGCTTCGGGACTCTACGACTCACGAGG
ACTCTCGACACTTACCGAAGCTTTGGGAATCTAGGACTTCCGACCCTTTACGATACTTTTGGACACTACGACTGTCGACA
ACTCCCGACACTTAACGATGCTTCAGGACCCTAACACTCACGACCCTTTGCGAACCTTTTGGACGCTACAACTGACGATT

ACTCGCGATACTTGTCGAACCTTCGGGACTCTATCACTTGCGATACTTTCCGAGCCTTAGGGAGCCTACTACTCTCGATT
ACTGGCGACACTTGTCGACACTTTTGGATGCTATTACTGACGAGTCTTTTCGACACTTGTGGACGCTACGACTGACGATC
ACTTGCGACTCTTCCCGAATCTTCGGGAGTCTACCACTTCCGACTCTTCCCGAAACTTCTGGAGTCTAAAACTTCCGATA
ACTCACGAAACTTACCGAATCTTCTGGAATCTAACACTTCCGAGTCTTTACGATCCTTTCGGATCCTAGCACTTACGATC
ACTGCCGAGTCTTACCGACTCTTAGGGAATCTATGACTCCCGAGCCTTCACGATGCTTGAGGACGCTATAACTCCCGAAC
ACTGACGAAACTTCCCGAGCCTTCCGGATCCTAGCACTAGCGAAGCTTCTCGATCCTTTTGGAATCTACTACTTTCGAGG
ACTCACGATCCTTTGCGACTCTTCTGGAGGCTAGCACTTTCGACACTTCTCGACACTTGCGGAACCTATTACTGCCGAGC
ACTGCCGAAACTTCCCGAACCTTCCGGAGTCTATAACTCACGACGCTTCACGACACTTATGGAAACTACCACTGCCGATT
ACTTACGAGTCTTGCCGAGTCTTCTGGAAGCTATTACTTTCGACCCTTTGCGATACTTCCGGACCCTATAACTCTCGAGA
ACTCACGAGTCTTGCCGATTCTTAAGGATTCTATTACTTTCGAGCCTTGGCGACGCTTTCGGAGGCTATAACTCCCGATA
ACTAGCGAGGCTTGGCGACTCTTTTGGAAGCTATCACTCCCGACTCTTGGCGAGTCTTTCGGATCCTAGAACTCGCGATT
ACTCTCGATCCTTTGCGATTCTTGCGGACCCTACGACTGTCGAAACTTTTCGACACTTTTGGATACTATCACTCCCGATC
ACTGGCGAGCCTTTCCGACGCTTACGGAGCCTAAAACTATCGAAACTTTTCGATACTTTTGGACCCTATCACTTGCGACT
ACTAGCGATCCTTCACGAATCTTTCGGAATCTATAACTAACGACTCTTCACGAAGCTTCTGGAGTCTAACACTCTCGACG
ACTCACGAACCTTTGCGATCCTTAGGGATACTAAGACTCACGAGACTTGGCGACTCTTGCGGAGGCTAGGACTTTCGAAG
ACTGACGACGCTTAACGATACTTAAGGAGTCTACAACTCCCGAGACTTCCCGATCCTTGAGGAGTCTATAACTATCGAGT
ACTCGCGAGTCTTGACGAGGCTTATGGAGGCTAACACTATCGATTCTTGTCGATACTTGAGGAACCTAACACTCGCGATT
ACTACCGACTCTTCCCGATCCTTAAGGATCCTACGACTCACGACACTTGGCGAACCTTATGGAGCCTAGCACTTCCGACC
ACTGGCGAATCTTAACGAATCTTGGGGAAGCTAGTACTGGCGATTCTTCCCGACACTTGTGGAGGCTAATACTCTCGACA
ACTCACGACCCTTCACGACACTTCGGGATGCTATTACTGCCGAGGCTTCACGACTCTTGGGGAGGCTAACACTTCCGAAC
ACTGTCGACACTTCGCGATACTTTTGGATACTATTACTGTCGAGCCTTATCGATGCTTGCGGAAACTAGTACTTACGACT
ACTCTCGAAACTTGACGAATCTTAGGGATCCTAACACTAGCGATTCTTTTCGATACTTATGGATTCTATTACTACCGACT
ACTTGCGACTCTTATCGAGACTTCCGGAGACTACCACTTGCGAGCCTTAGCGACCCTTCAGGATACTATAACTGACGAGT
ACTTACGATACTTGACGATCCTTTCGGAACCTAATACTTGCGAGTCTTCGCGACCCTTCTGGATACTATCACTTTCGAGC
ACTTCCGATGCTTCGCGACCCTTTCGGACCCTACTACTGACGACCCTTCCCGAAGCTTTCGGAGACTACCACTTCCGATG
ACTTACGATTCTTTGCGAGTCTTTTGGAGGCTAGCACTTTCGAATCTTCACGATTCTTGTGGATCCTACTACTTACGATG
ACTCGCGATTCTTAACGATACTTCAGGATACTACAACTTACGACTCTTATCGACTCTTCGGGACGCTAGTACTCACGAGC
ACTGGCGAGGCTTGCCGAGCCTTCTGGATTCTAAAACTGACGATTCTTCTCGATTCTTGCGGAGTCTACTACTCACGACT
ACTTCCGACCCTTCACGAAACTTTAGGACACTATCACTGCCGAACCTTGCCGACTCTTGCGGAATCTACGACTATCGATT
ACTTTCGACTCTTTTCGAAACTTTCGGAGGCTAAAACTGCCGACTCTTCACGATTCTTGAGGAAGCTATGACTTACGAAA
ACTAACGACGCTTCACGACTCTTCTGGATGCTAGTACTCGCGACCCTTTACGAACCTTACGGATACTAGAACTGGCGATC
ACTATCGATTCTTACCGACTCTTATGGACTCTACCACTCCCGACCCTTTGCGAGCCTTGCGGAATCTATCACTACCGAGT
ACTTGCGAGACTTTGCGATTCTTATGGATACTACAACTCACGACCCTTGGCGAACCTTGTGGATACTACTACTGGCGATT
ACTGACGAGGCTTGCCGAACCTTTCGGAGTCTAGGACTCCCGAATCTTCACGATTCTTCGGGAAGCTATAACTATCGAGC
ACTACCGAGCCTTAGCGACGCTTGGGGATACTACTACTGCCGAGTCTTTGCGAGTCTTCAGGACGCTAACACTGACGAGG
ACTAACGATTCTTCCCGATTCTTCAGGATGCTACCACTTGCGAGACTTGACGACGCTTGAGGAATCTACCACTCTCGAAG
ACTCGCGAACCTTCTCGACGCTTTCGGACCCTAAAACTATCGAAACTTGCCGATTCTTATGGATTCTAAGACTCTCGAAC
ACTCCCGAAGCTTTTCGATACTTCAGGATTCTACGACTCCCGACGCTTTCCGAGTCTTGGGGAAACTAGCACTGTCGATC
ACTCTCGATTCTTAACGAGCCTTTGGGACACTACCACTTCCGAGTCTTGCCGAGCCTTTCGGATTCTATCACTCTCGACG
ACTAACGAACCTTCCCGACCCTTTTGGAGTCTAACACTATCGACACTTTTCGATCCTTTTGGAGCCTAGCACTTCCGAAT
ACTCCCGACCCTTTTCGACACTTTGGGAGTCTATAACTTCCGACGCTTCGCGATCCTTCCGGATGCTAGAACTTACGACA
ACTGTCGACTCTTGCCGAAGCTTCCGGAAACTAACACTTACGAGTCTTTGCGAGACTTAGGGATTCTATGACTGGCGAGT
ACTAGCGACACTTTGCGATTCTTCGGGATCCTATTACTTGCGACCCTTTTCGAGTCTTAGGGAAACTATTACTCTCGATT
ACTCCCGATGCTTTTCGATTCTTTGGGATGCTACCACTCGCGATGCTTCGCGAGCCTTTCGGAGTCTAGTACTGTCGAGA
ACTGCCGATTCTTTACGAACCTTTCGGACGCTAGTACTAACGACCCTTTGCGAGTCTTATGGAACCTATCACTGCCGACG
ACTTCCGAGACTTTCCGATACTTCCGGACCCTACGACTATCGACCCTTTACGATACTTGCGGAATCTACTACTTACGACT
ACTAACGAGTCTTACCGAAGCTTTTGGATGCTACTACTCCCGACTCTTACCGACTCTTGGGGAGCCTAAGACTACCGATT
ACTGCCGAATCTTATCGACGCTTCGGGACTCTACAACTAACGACGCTTATCGACCCTTTCGGAAACTATTACTAACGAGC
ACTTTCGATTCTTCACGAAGCTTTGGGATGCTAAGACTGGCGATGCTTTCCGACTCTTCAGGAACCTACAACTCACGAGG
ACTACCGACTCTTTGCGAGTCTTGCGGAGGCTATAACTGACGACTCTTATCGAGTCTTCGGGACTCTAATACTGCCGACT
ACTCCCGATCCTTGACGACACTTTAGGATGCTAGAACTAACGATTCTTGACGACACTTCGGGACTCTACAACTTGCGACT
ACTAGCGAATCTTCGCGACTCTTAAGGATCCTATTACTTACGACTCTTGTCGAATCTTTCGGACCCTATCACTGCCGACG
ACTGCCGACGCTTGGCGAGGCTTTAGGACGCTAGAACTCACGAGACTTTACGAGTCTTGTGGACGCTATCACTATCGAGA
ACTTACGATACTTCTCGACCCTTACGGAATCTAATACTTACGATTCTTTTCGAGGCTTCAGGAAGCTAGGACTGACGATC
ACTTACGAAACTTATCGAAACTTCGGGAGGCTAACACTGGCGAGACTTTTCGACACTTAAGGATACTAGAACTGTCGATC
ACTAGCGAACCTTTGCGATTCTTGAGGACCCTAATACTCTCGATACTTAGCGATCCTTGAGGAGTCTACTACTCTCGATC
ACTGACGACCCTTTTCGAGTCTTTGGGACGCTATGACTGTCGACTCTTAGCGAGCCTTTTGGAACCTATGACTTCCGATT
ACTACCGATCCTTTACGAAACTTCCGGAGACTACGACTCACGATACTTCACGAGACTTCGGGATCCTAATACTGCCGACG
ACTCCCGACCCTTGTCGAGTCTTTTGGACACTATCACTGGCGAGTCTTCCCGACCCTTATGGATTCTATTACTTACGATC
ACTTCCGATTCTTTACGACACTTGTGGAACCTACAACTCGCGACTCTTGGCGAGACTTCAGGACTCTACCACTTTCGACG
ACTATCGAAACTTAGCGAAACTTCCGGATTCTAACACTAGCGACTCTTCACGATCCTTTGGGACTCTACAACTTTCGACG
ACTATCGACTCTTGCCGAGACTTAAGGAAGCTAGTACTAGCGAGGCTTTGCGATCCTTCTGGAACCTATCACTCTCGAAA

ACTCACGAATCTTTACGAGCCTTCGGGAGCCTATCACTGGCGAATCTTGCCGAAGCTTTCGGAGGCTATCACTTTCGACT
ACTGGCGAGGCTTGACGATTCTTCCGGAAACTACAACTTACGATGCTTTTCGAAGCTTACGGATTCTAATACTCCCGATT
ACTAGCGAGACTTCACGAGTCTTGCGGAAGCTAGCACTATCGACTCTTCACGATCCTTTAGGAACCTATTACTACCGACA
ACTTGCGACTCTTAGCGAGTCTTCTGGACTCTACCACTTACGAGACTTTCCGACTCTTCTGGAGTCTAGTACTGCCGAAC
ACTTTCGAAGCTTTCCGAGGCTTATGGAGACTATGACTCACGAATCTTTTCGATACTTAAGGATACTATAACTATCGATT
ACTGACGATCCTTCCCGAGTCTTTTGGAGCCTAACACTTACGAGACTTCCCGAACCTTCCGGATGCTAGGACTTACGAAG
ACTCGCGAAACTTCTCGACGCTTACGGATTCTATTACTCACGACTCTTAACGAATCTTTCGGACGCTACCACTTCCGAAA
ACTTCCGATCCTTGCCGAAGCTTCCGGAGGCTAGCACTCACGAAGCTTTACGATTCTTAAGGATACTAAGACTTCCGACT
ACTATCGACTCTTTCCGATGCTTTCGGAGCCTATAACTCCCGACGCTTCGCGAAACTTCGGGAGACTAAAACTTCCGACG
ACTTTCGAGTCTTAACGATCCTTTGGGAACCTACGACTCGCGATACTTTGCGACTCTTTGGGATTCTATTACTTCCGACT
ACTTTCGAGCCTTTACGACGCTTGAGGACTCTAGCACTGCCGATCCTTGTCGATACTTACGGAGCCTACCACTGACGATT
ACTTCCGAATCTTTTCGACCCTTGGGGAATCTACGACTTACGACTCTTGACGAAACTTTCGGACCCTAGTACTACCGATG
ACTGTCGATGCTTTACGAGGCTTCAGGATACTAGTACTCCCGACCCTTGGCGACTCTTCCGGATGCTAGTACTTACGAAG
ACTATCGATGCTTAACGAGACTTGGGGAACCTAAGACTAGCGACGCTTCGCGAACCTTGTGGAAGCTAGCACTCGCGACT
ACTGCCGAGTCTTCCCGAGGCTTTAGGAACCTACTACTAACGACCCTTTTCGACCCTTTGGGACCCTATTACTGCCGACG
ACTTTCGATCCTTCACGACGCTTCCGGACTCTAATACTGACGATTCTTTACGATTCTTACGGAGTCTAGCACTGTCGAAC
ACTCACGAGCCTTTGCGACTCTTATGGAACCTATTACTGTCGACACTTGCCGATCCTTCTGGAGGCTACGACTGTCGACG
ACTTGCGATACTTCGCGAATCTTCCGGAGACTATGACTGCCGATCCTTACCGAGGCTTGCGGAACCTATTACTACCGATC
ACTATCGAGCCTTGCCGATGCTTATGGAGTCTAAAACTCCCGACGCTTGCCGACGCTTGCGGATCCTAAAACTCGCGATC
ACTAACGACGCTTATCGACTCTTCGGGATGCTATTACTCGCGACGCTTTCCGACCCTTAGGGACACTAGAACTTCCGATG
ACTGTCGAGGCTTCGCGAATCTTTAGGAATCTAATACTTCCGAGCCTTGCCGAGGCTTTCGGAGTCTACCACTACCGATT
ACTTCCGAGGCTTTCCGATTCTTCCGGAGTCTACAACTCGCGATTCTTCGCGAGCCTTAAGGACACTAGGACTAGCGACT
ACTAGCGACCCTTATCGAGTCTTCCGGATCCTATCACTGTCGATGCTTCCCGAGACTTCGGGAACCTACTACTTGCGATC
ACTGACGACGCTTCACGAGCCTTCCGGAAGCTATTACTATCGAATCTTTGCGATGCTTTTGGAAGCTATAACTCCCGACA
ACTCTCGATTCTTCACGAACCTTTTGGATCCTATTACTTGCGAAGCTTGCCGACTCTTGGGGAGGCTACAACTCGCGAGC
ACTCGCGAGACTTCACGAGACTTTGGGACTCTATTACTTACGATTCTTCGCGAGTCTTCAGGACTCTACGACTGCCGATC
ACTCGCGAATCTTGGCGAGCCTTTGGGATCCTAGCACTTTCGAAACTTATCGAGCCTTCTGGAGGCTACGACTAGCGACC
ACTTTCGAACCTTTTCGAGCCTTGTGGACCCTAACACTTACGACGCTTGTCGATCCTTCCGGACCCTATAACTTGCGACC
ACTAACGACCCTTTTCGATCCTTCCGGAGGCTAAAACTTCCGATTCTTATCGAAGCTTCTGGATACTAGTACTCTCGACC
ACTCCCGAAGCTTGACGAATCTTGAGGACTCTAGCACTCGCGACGCTTTTCGAGACTTGCGGACTCTAGCACTTTCGAGC
ACTTTCGAGACTTCACGATACTTTCGGAAGCTAAAACTTTCGACTCTTTGCGATCCTTAAGGAGCCTATAACTTTCGAAT
ACTATCGATGCTTGGCGAGTCTTATGGAGCCTAGGACTGCCGAGGCTTGCCGACGCTTATGGAATCTACGACTTGCGATC
ACTCACGACTCTTGCCGAATCTTGTGGACACTACAACTTACGACGCTTTTCGATTCTTATGGATTCTATTACTAACGACC
ACTCCCGATCCTTCTCGACTCTTTTGGAAACTAAAACTATCGAACCTTTGCGATGCTTTGGGAACCTACAACTGGCGAGT
ACTGCCGAAACTTTCCGACGCTTTTGGATTCTAAGACTCCCGAAGCTTTCCGAGTCTTATGGAGACTACTACTTCCGACT
ACTGTCGAGCCTTGGCGACACTTGCGGATTCTAACACTGGCGAGACTTTGCGATCCTTAAGGAATCTACTACTTTCGAAA
ACTCGCGACCCTTTACGAGTCTTTGGGACTCTACCACTATCGAAACTTTACGATGCTTTCGGATTCTAGTACTCCCGATC
ACTTTCGAGACTTTGCGAGGCTTCGGGATGCTAAAACTATCGATGCTTATCGATTCTTTAGGAGACTATTACTCTCGATC
ACTCACGAGGCTTAACGATTCTTGTGGACTCTATCACTCCCGACTCTTCACGAAGCTTAGGGACCCTAATACTCTCGAGA
ACTCTCGATACTTCTCGATGCTTCGGGAACCTATTACTTACGAACCTTCTCGATTCTTCAGGAATCTACCACTTACGATC
ACTCGCGATGCTTTACGATACTTACGGAATCTAGGACTCTCGAAGCTTAACGACGCTTGCGGACACTAGGACTTCCGACA
ACTATCGATGCTTTACGACCCTTCAGGATTCTATCACTCGCGACACTTCCCGACTCTTGTGGATCCTATAACTCGCGAGA
ACTCTCGATACTTGACGAGCCTTGGGGAATCTAGCACTGTCGATACTTTGCGACCCTTGGGGACACTATGACTACCGATG
ACTGTCGAAGCTTTACGATGCTTCCGGACACTAGAACTTGCGACACTTAGCGAATCTTATGGATGCTAGCACTTACGATG
ACTTCCGATTCTTTACGAATCTTCCGGACGCTAACACTCCCGAAGCTTGGCGAACCTTTTGGATTCTACTACTTTCGATC
ACTGGCGAGCCTTGTCGAAGCTTGCGGAGTCTAAAACTGACGACTCTTCTCGACGCTTACGGAACCTACTACTTTCGACG
ACTCTCGATCCTTCGCGACTCTTGGGGATTCTAACACTGTCGAACCTTGTCGAAACTTCAGGAAACTAATACTTACGAGG
ACTGGCGAGACTTTGCGATCCTTCTGGATCCTATTACTTTCGAGACTTTACGAATCTTGGGGATACTATTACTAACGAGT
ACTTGCGATTCTTCACGAGGCTTAAGGATCCTAATACTTTCGAGCCTTAGCGACGCTTATGGAGCCTACCACTGGCGACC
ACTACCGAGTCTTCTCGACGCTTCTGGACACTAATACTTCCGAAGCTTGCCGAGACTTGTGGAAACTAGCACTCTCGATC
ACTTTCGAATCTTGTCGAAACTTTAGGACTCTACCACTAGCGACTCTTAGCGATCCTTTTGGATACTATTACTCACGAGT
ACTGCCGACCCTTCACGAAACTTGAGGACTCTATTACTAGCGACACTTAGCGAGTCTTAAGGAACCTACTACTCTCGACG
ACTTGCGAAGCTTGACGAAACTTGGGGAGCCTATAACTCCCGAGTCTTGGCGAGCCTTTAGGAGTCTACTACTCCCGAAA
ACTCACGAGGCTTAACGATTCTTATGGAGTCTACTACTGACGAATCTTGACGAAACTTCGGGACTCTATTACTTCCGATA
ACTGCCGACACTTGACGAGTCTTTCGGAACCTAACACTGGCGACTCTTTCCGACGCTTCCGGAGGCTATTACTCTCGACA
ACTCGCGACTCTTCTCGAAACTTCCGGATTCTATAACTATCGAAGCTTATCGAGACTTTTGGACCCTAACACTCTCGAGA
ACTCCCGAGGCTTAACGAAGCTTGGGGAGGCTAGAACTATCGACTCTTTTCGATACTTTTGGACTCTATCACTTTCGACC
ACTGCCGAGTCTTTTCGATCCTTATGGAGGCTACCACTATCGATGCTTGACGATGCTTTAGGAACCTATTACTGTCGATG
ACTCCCGACTCTTTACGACCCTTTGGGAGGCTACAACTTGCGAGTCTTGCCGACTCTTCCGGAGACTAATACTCACGACT
ACTCTCGATTCTTTACGATTCTTGGGGATCCTATGACTTTCGAATCTTTCCGACTCTTCAGGATTCTATTACTGTCGATC
ACTACCGAGGCTTTTCGACTCTTTCGGAATCTAGTACTATCGAACCTTCCCGATACTTCCGGAAACTATAACTTCCGAGA
ACTGCCGATTCTTACCGACACTTGAGGAAACTAAGACTCACGAGGCTTCTCGATTCTTGTGGACGCTAGTACTGTCGATC

ACTGGCGATTCTTCGCGACCCTTGAGGAGCCTATGACTAACGACACTTGGCGAACCTTTTGGAGCCTAGTACTCACGATC
ACTAGCGACTCTTACCGACACTTTGGGAATCTAGGACTCGCGATGCTTATCGAGTCTTCGGGACTCTACTACTGGCGATT
ACTTCCGAATCTTTTCGAGCCTTGGGGAAACTAATACTAGCGACGCTTGTCGAGACTTTTGGACACTAAAACTCTCGACG
ACTAACGATGCTTCTCGATACTTACGGAGTCTACCACTATCGATTCTTGCCGAGGCTTAAGGAGTCTAATACTCTCGAGA
ACTTGCGATGCTTATCGACGCTTGAGGATGCTATCACTGTCGACACTTTTCGATCCTTACGGACGCTATCACTTACGACA
ACTTGCGATCCTTAACGACGCTTGTGGACCCTACCACTAGCGATTCTTCGCGATTCTTCAGGATTCTATCACTTTCGATT
ACTCCCGAGCCTTCACGAGGCTTTGGGAAACTACGACTTTCGAATCTTGTCGATGCTTTCGGATGCTACGACTAGCGAAG
ACTTCCGAAGCTTAGCGACCCTTCAGGACCCTATCACTCCCGATCCTTCCCGATTCTTCCGGACACTAAAACTCCCGAGA
ACTCGCGACTCTTGTCGATGCTTAAGGACCCTACCACTACCGACTCTTCGCGAAACTTGCGGACGCTATCACTTTCGAAA
ACTGTCGAATCTTAGCGAAACTTTCGGATCCTAGCACTGCCGATGCTTTTCGATACTTGTGGAAACTACGACTTGCGACC
ACTCACGAAACTTGGCGACTCTTTGGGACCCTAATACTTGCGATTCTTCTCGATCCTTCGGGACACTATTACTCACGAAC
ACTCGCGATTCTTTGCGACACTTTTGGAAACTATTACTTACGAGTCTTCACGATGCTTATGGAGTCTATCACTTTCGAGG
ACTGTCGAGCCTTCCCGAAGCTTCAGGAAACTAACACTGGCGACGCTTCGCGACACTTTTGGATTCTACAACTATCGACT
ACTACCGAACCTTCACGAGACTTTAGGAGACTAAGACTGTCGATGCTTGCCGATCCTTGGGGAGGCTATAACTGGCGAGT
ACTTTCGAGGCTTGACGACCCTTACGGAGCCTATGACTGACGACCCTTTTCGACGCTTCGGGATTCTAGCACTTGCGAGC
ACTTCCGATTCTTCTCGATACTTCTGGAGGCTACTACTTTCGAGGCTTGGCGATGCTTACGGAGCCTATAACTGGCGAGG
ACTCCCGAATCTTGGCGACTCTTGCGGAAGCTATCACTAACGATTCTTACCGATGCTTACGGATACTACCACTTGCGACC
ACTGGCGACGCTTATCGACTCTTTAGGATCCTACCACTTCCGAGTCTTAGCGACTCTTAAGGAGCCTACTACTTTCGATC
ACTCGCGATGCTTCACGATACTTTCGGAAACTAGAACTTTCGAGTCTTAACGAGACTTGCGGAAGCTACTACTCGCGACA
ACTAACGAAGCTTCCCGAAGCTTTTGGACCCTACTACTAGCGACTCTTCGCGATTCTTCTGGATACTACGACTGACGAGC
ACTCACGATTCTTTGCGAGTCTTCGGGATCCTATCACTCGCGACCCTTGGCGAGACTTTTGGAGACTACAACTTACGATG
ACTGCCGAGTCTTTTCGACACTTTGGGAGACTAGAACTTACGAAGCTTATCGAGCCTTATGGACGCTACCACTACCGATC
ACTTACGACTCTTGGCGAGTCTTTTGGACCCTATAACTGCCGACGCTTGTCGATCCTTTTGGACCCTATCACTTCCGAAG
ACTCGCGACCCTTAGCGATGCTTGTGGAAACTAAGACTACCGAGTCTTGACGAGACTTTTGGACCCTACCACTCGCGACA
ACTACCGACACTTCACGATCCTTCCGGATACTAATACTCACGAGCCTTCCCGATACTTATGGACTCTAGTACTTGCGAGC
ACTTGCGAGACTTAACGACCCTTTTGGAAACTATCACTACCGATACTTTTCGATTCTTGCGGACTCTACCACTCGCGAAC
ACTACCGAACCTTCTCGATCCTTGGGGAGTCTATGACTTACGACGCTTTGCGAGTCTTTAGGATCCTACGACTCACGACC
ACTTACGATTCTTCTCGAAACTTTTGGACACTACGACTATCGACTCTTTCCGATTCTTTGGGAAACTACTACTCTCGACC
ACTTGCGACACTTAACGACTCTTACGGAAACTATTACTCGCGAGACTTGTCGAAACTTTTGGACTCTAGCACTGGCGACT
ACTGACGAATCTTTTCGAATCTTTTGGAGTCTAGCACTGACGAGCCTTTACGATACTTCTGGAACCTATAACTCACGACG
ACTTCCGAACCTTCGCGACCCTTAGGGATTCTACCACTGACGAGCCTTTGCGAAACTTTAGGATCCTACTACTTACGATT
ACTCGCGACCCTTTACGATGCTTTTGGAGGCTATCACTGACGAATCTTGGCGAGCCTTCCGGAAGCTAAGACTCCCGATC
ACTGCCGATGCTTGGCGACCCTTACGGAAGCTACAACTGTCGACCCTTCTCGACACTTTCGGAACCTACAACTACCGATC
ACTCCCGACACTTCGCGAACCTTAAGGATCCTAATACTCGCGATTCTTTTCGAAGCTTTAGGAGACTATCACTAGCGATC
ACTCTCGACTCTTCGCGATTCTTATGGAATCTAAAACTTTCGATCCTTCCCGACCCTTACGGATCCTAGTACTTTCGATT
ACTACCGACGCTTCCCGATCCTTCGGGATCCTACCACTTCCGACCCTTCGCGATCCTTAAGGACCCTAATACTACCGATC
